# Structure of a bacterial α-1,2-glucosidase defines mechanisms of hydrolysis and substrate specificity in GH65 family hydrolases

**DOI:** 10.1016/j.jbc.2021.101366

**Published:** 2021-10-30

**Authors:** Shuntaro Nakamura, Takanori Nihira, Rikuya Kurata, Hiroyuki Nakai, Kazumi Funane, Enoch Y. Park, Takatsugu Miyazaki

**Affiliations:** 1 Department of Bioscience, Graduate School of Science and Technology, Shizuoka University, Shizuoka, Japan; 2 Faculty of Agriculture, Niigata University, Niigata, Japan; 3 Department of Agriculture, Graduate School of Integrated Science and Technology, Shizuoka University, Shizuoka, Japan; 4 Faculty of Life and Environmental Sciences, University of Yamanashi, Kofu, Yamanashi, Japan; 5 Research Institute of Green Science and Technology, Shizuoka University, Shizuoka, Japan

**Keywords:** α-1,2-glucosidase, glycoside hydrolase family 65, kojibiose, dextran, *Flavobacterium johnsoniae*, crystal structure, glycoside hydrolase, polysaccharide utilization system, oligosaccharide, enzyme mechanism, α-GlcF, α-D-glucopyranosyl fluoride, BsGGP, 2-*O*-α-glucosylglycerol phosphorylase from *Bacillus selenitireducens*, CAZymes, carbohydrate-active enzymes, CsKP, kojibiose phosphorylase from *Caldicellulosiruptor saccharolyticus*, G2G2G6G, 6-*O*-α-kojitriosylglucose, G2G6G, 6-*O*-α-kojibiosylglucose, GH, glycoside hydrolases, GH65, glycoside hydrolase family 65, GPs, glycoside phosphorylases, Hyl, hydroxylysine, LbMP, maltose phosphorylase from *Levilactobacillus brevis*, PGGHG, Protein α-glucosyl-1,2-β-galactosyl-L-hydroxylysine α-glucosidase, TtGA, glucoamylase from *Thermoanaerobacterium themosaccharolyticum*

## Abstract

Glycoside hydrolase family 65 (GH65) comprises glycoside hydrolases (GHs) and glycoside phosphorylases (GPs) that act on α-glucosidic linkages in oligosaccharides. All previously reported bacterial GH65 enzymes are GPs, whereas all eukaryotic GH65 enzymes known are GHs. In addition, to date, no crystal structure of a GH65 GH has yet been reported. In this study, we use biochemical experiments and X-ray crystallography to examine the function and structure of a GH65 enzyme from *Flavobacterium johnsoniae* (FjGH65A) that shows low amino acid sequence homology to reported GH65 enzymes. We found that FjGH65A does not exhibit phosphorolytic activity, but it does hydrolyze kojibiose (α-1,2-glucobiose) and oligosaccharides containing a kojibiosyl moiety without requiring inorganic phosphate. In addition, stereochemical analysis demonstrated that FjGH65A catalyzes this hydrolytic reaction *via* an anomer-inverting mechanism. The three-dimensional structures of FjGH65A in native form and in complex with glucose were determined at resolutions of 1.54 and 1.40 Å resolutions, respectively. The overall structure of FjGH65A resembled those of other GH65 GPs, and the general acid catalyst Glu^472^ was conserved. However, the amino acid sequence forming the phosphate-binding site typical of GH65 GPs was not conserved in FjGH65A. Moreover, FjGH65A had the general base catalyst Glu^616^ instead, which is required to activate a nucleophilic water molecule. These results indicate that FjGH65A is an α-1,2-glucosidase and is the first bacterial GH found in the GH65 family.

Glucose is the most abundant monosaccharide in nature, and its oligomers and polymers have various properties and physiological functions. For example, starch, which is a glucose polymer with α-1,4- and α-1,6-linkages, serves as an energy storage material in plants, whereas glucose residues play an important role in protein quality control during the processing of eukaryotic *N*-glycans (1, 2). Carbohydrate active enzymes (CAZymes) are involved in the biosynthesis and degradation of diverse carbohydrates including glucosides and are classified based on amino acid sequence homology: these include various families of glycoside hydrolases (GHs), glycosyltransferases, polysaccharide lyases, carbohydrate esterases, and auxiliary activities that have been established and registered in the CAZy database (http://www.cazy.org/) (3, 4, 5). GHs are divided into the largest number of families in the CAZy database, with 171 families established as of August 2021 (several families are now considered obsolete). Some GH families have been further grouped into clans (GH-A to GH-R) on the basis of their structural similarity and catalytic mechanism (6). Although many enzymes with various substrate specificities have been reported in GH families, there is still an abundance of putative GHs with unknown functions.

The glycoside hydrolase family 65 (GH65) is composed of GHs and glycoside phosphorylases (GPs) acting on α-glucosidic linkages in oligosaccharides and polysaccharides. Of more than 160 GH families, GPs are found in GH families such as GH3, GH13, GH65, GH94, GH112, GH130, GH149, and GH161. Of these, GH65 (7), GH94 (8), GH112 (9), GH130 (10, 11), GH149 (12), and GH161 (13) are classified as anomer-inverting GPs that are active against various substrates; the inverting GPs except for GH65 GPs are active on β-glycosides.

All GH65 GPs have been found in bacteria and catalyze phosphorolysis. The reaction mechanism of the GH65 GPs has been proposed to be similar to the inverting hydrolytic mechanism that uses a general acid and a general base as catalytic residues, that is, the single-displacement mechanism (Fig. 1*A*). In GH65 GPs, a phosphate nucleophile attack on the glycosidic bond of the substrate is assisted by proton donation from a general acid catalyst to the glycosidic oxygen (Fig. 1*B*). The inverting GPs also catalyze reverse phosphorolysis to form glycosidic bonds (14). The GH65 GPs that are reported to be active on α-glucobioses, such as trehalose (α-1,1) (15, 16), kojibiose (α-1,2) (17, 18, 19), nigerose (α-1,3) (20, 21), and maltose (α-1,4) (22, 23), produce β-glucose 1-phosphate and glucose. The other known GH65 GPs include trehalose-6-phosphate phosphorylase (24), 3-*O*-α-D-glucopyranosyl-L-rhamnose phosphorylase (25), 2-*O*-glucopyranosylglycerol phosphorylase (26), and α-1,3-oligo-D-glucan phosphorylase (21). The common structure of these GH65 GPs has been identified and comprises four regions as follows: an N-terminal β-sandwich domain, a helical linker, an (α/α)_6_-barrel catalytic domain, and a C-terminal β-sheet domain (14). Based on this structure, two consecutive serine residues essential for phosphate binding and the general acid residues have been identified in GH65 GPs; the catalytic mechanism is also well understood (27, 28).

**Figure 1:**
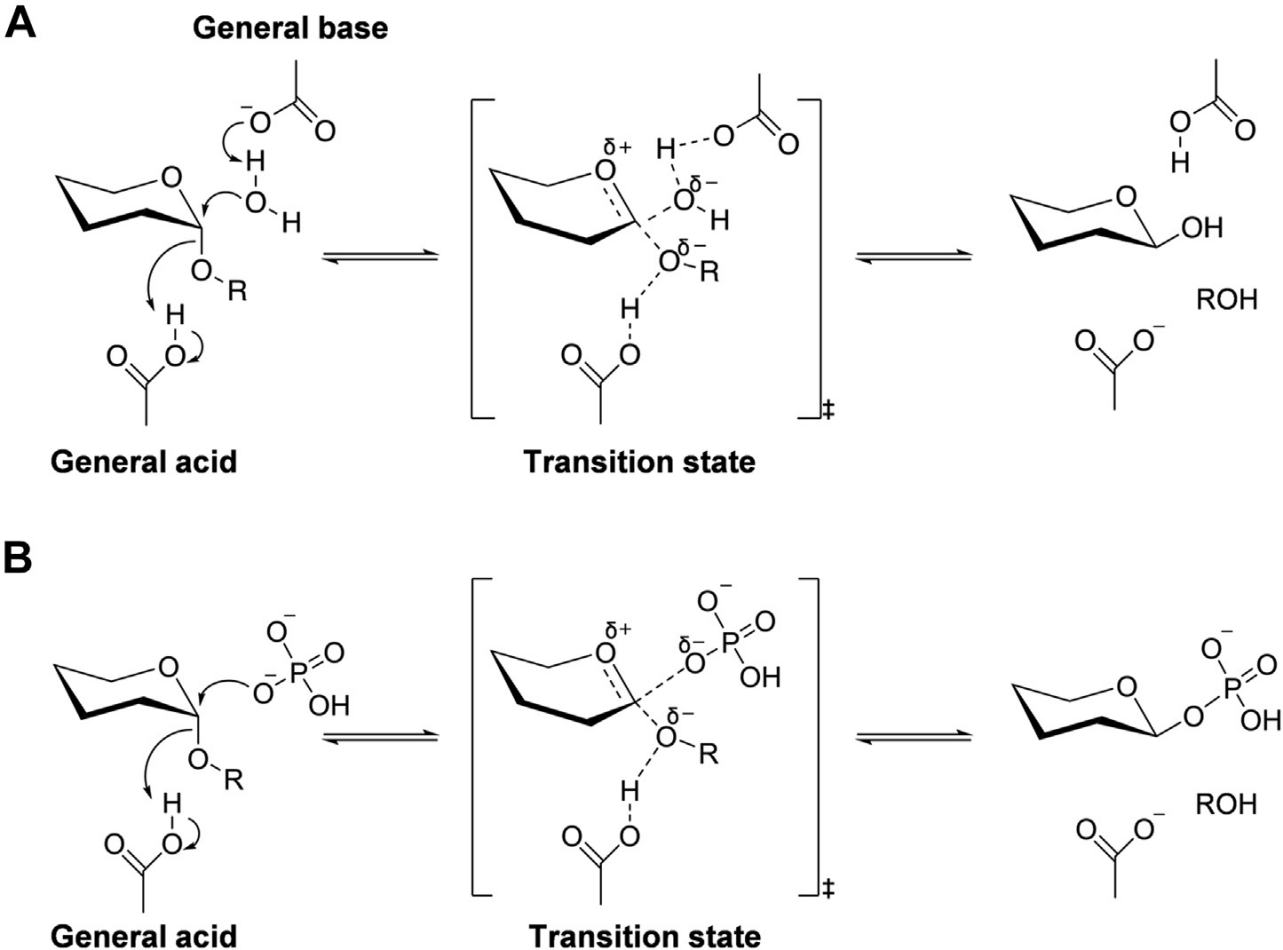
**Catalytic mechanisms for inverting glycoside phosphorylase and glycoside hydrolase.***A*, the reaction mechanism of inverting α-glycoside hydrolase. *B*, the reaction mechanism of inverting α-glycoside phosphorylase.

Conversely, GH65 GHs have been found only in eukaryotes, and to date, only two activities have been reported. Acid trehalases, found in fungi, are active under acidic conditions, but their biochemical properties are not clear (29, 30). Protein α-glucosyl-1,2-β-galactosyl-L-hydroxylysine α-glucosidase (PGGHG) was found in *Homo sapiens* and *Gallus gallus* approximately 40 years ago and releases glucose from a disaccharide unit (2-*O*-glucopyranosyl galactopyranose, Glc-α1,2-Gal) attached to a hydroxylysine (Hyl) residue of collagen (31). The genes for PGGHGs were recently identified and classified as members of the GH65 family, and recombinant enzymes showed hydrolytic activity against Glc-α1,2-Gal-Hyl and type IV collagen (32). No crystal structure of GH65 GHs has yet been reported, and their catalytic mechanism is still unknown.

*Flavobacterium johnsoniae* is a Gram-negative soil bacterium whose genome has been sequenced, an analysis of which has revealed that it possesses several putative GHs to degrade polysaccharides (33). In this study, we report a GH65 enzyme found in this bacterium that has a low amino acid sequence identity with other reported GH65 enzymes. This enzyme is not a GP but showed α-1,2-glucosidase activity, and its crystal structure, reported here, is the first structure from the GH65 GHs. This report therefore provides structural insight into substrate specificity and the catalytic mechanism of the GH65 GHs.

## Results

### Biochemical characterization of recombinant FjGH65A

**Figure 2:**
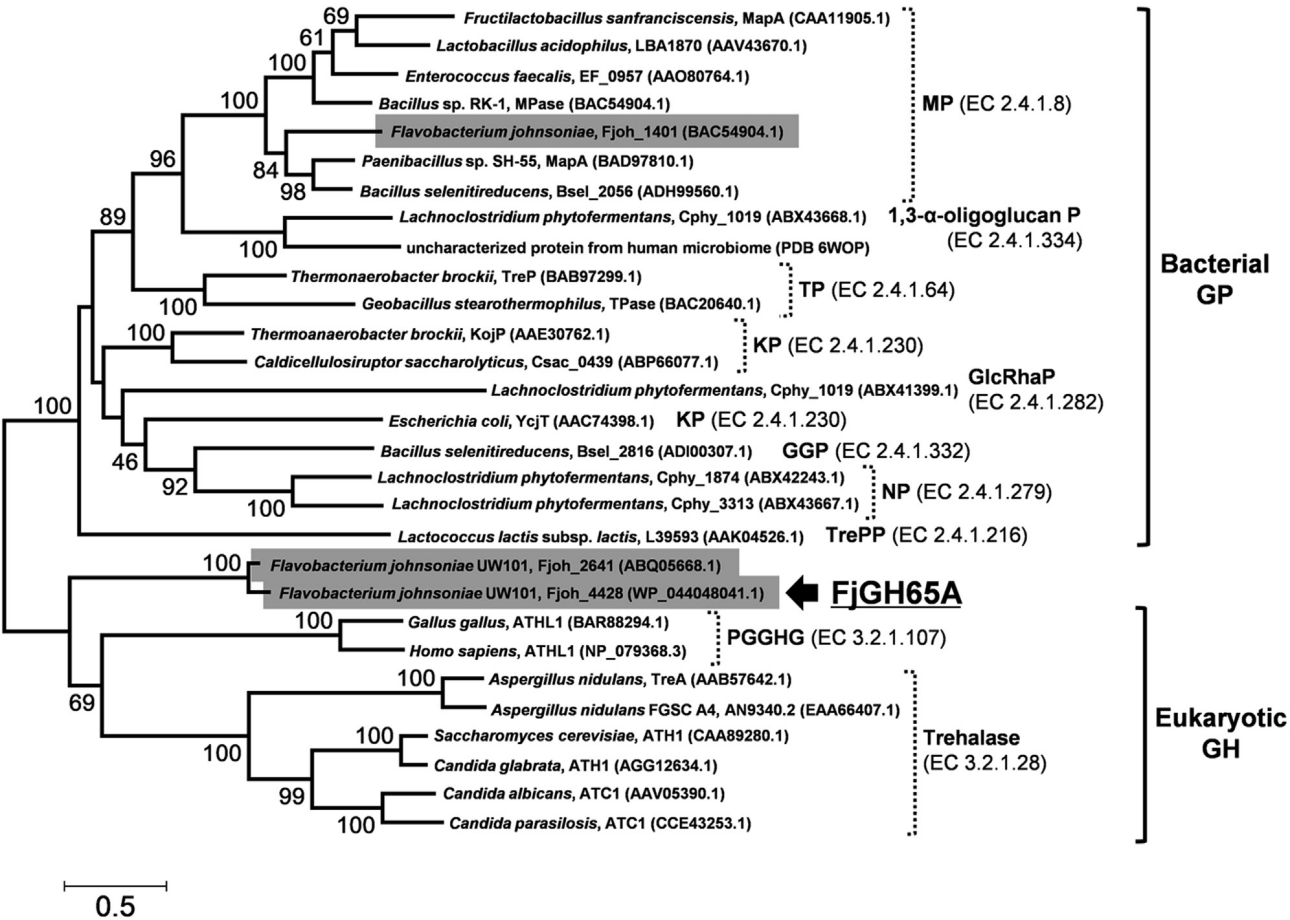
**Phylogenetic tree of the characterized enzymes and *Flavobacterium johnsoniae* proteins belonging to GH65.** The multiple sequence alignment was performed using MUSCLE (76), and the phylogenetic tree was constructed using the maximum likelihood method and was visualized using MEGA 7 (77). Amino acid sequences of all the enzymes were obtained from GenBank. Bootstrap values based on 1000 replicates are shown. Origins, protein names, and GenBank ID in parentheses are labeled for each branch. For the uncharacterized GH65 protein from the human microbiome, a Protein Data Bank (PDB) code is shown because its structure was only published in PDB. GH65 proteins from *F. johnsoniae* are highlighted in *gray boxes*. 1,3-α-oligoglucan P, 1,3-α-oligo-D-glucan phosphorylase; GGP, 2-*O*-α-glucosylglycerol phosphorylase; GH, glycoside hydrolase; GH65, glycoside hydrolase family 65; GlcRhaP, 3-*O*-α-D-glucopyranosyl-L-rhamnose phosphorylase; KP, kojibiose phosphorylase; MP, maltose phosphorylase; NP, nigerose phosphorylase; PGGHG, α-glucosyl-1,2-β-galactosyl-L-hydroxylysine α-glucosidase; TP, trehalose phosphorylase; TrePP, trehalose-6-phosphate phosphorylase.

*F. johnsoniae* possesses three genes for GH65 proteins, namely, Fjoh_1401, Fjoh_2641, and Fjoh_4428. A phylogenetic tree shows that Fjoh_2641 and Fjoh_4428 are included in a different clade from the bacterial GPs and eukaryotic GHs, whereas Fjoh_1401 belongs to the maltose phosphorylase clade (Fig. 2). Fjoh_4428 has lower than 33% sequence identity to known GH65 enzymes, including the GPs and GHs, and Fjoh_2641 lacks 288 amino acid residues at the N-terminal compared with Fjoh_4428. In this study, we therefore decided to characterize the function and structure of Fjoh_4428 (hereafter FjGH65A). FjGH65A without the N-terminal signal peptide was produced as a His_6_-tag-fused protein in *Escherichia coli* BL21 (DE3), and approximately 10 mg of purified FjGH65A was obtained from a cell lysate from a 500 ml culture. The theoretical molecular weight of FjGH65A was 76.4 kDa, which is consistent with the size of a single band for FjGH65A on an SDS-PAGE (Fig. S1*A*). Gel filtration chromatography showed that the molecular weight of FjGH65A was 433 ± 0.92 kDa, suggesting that this protein was a hexamer in solution (Fig. S1*B*).

**Figure 3:**
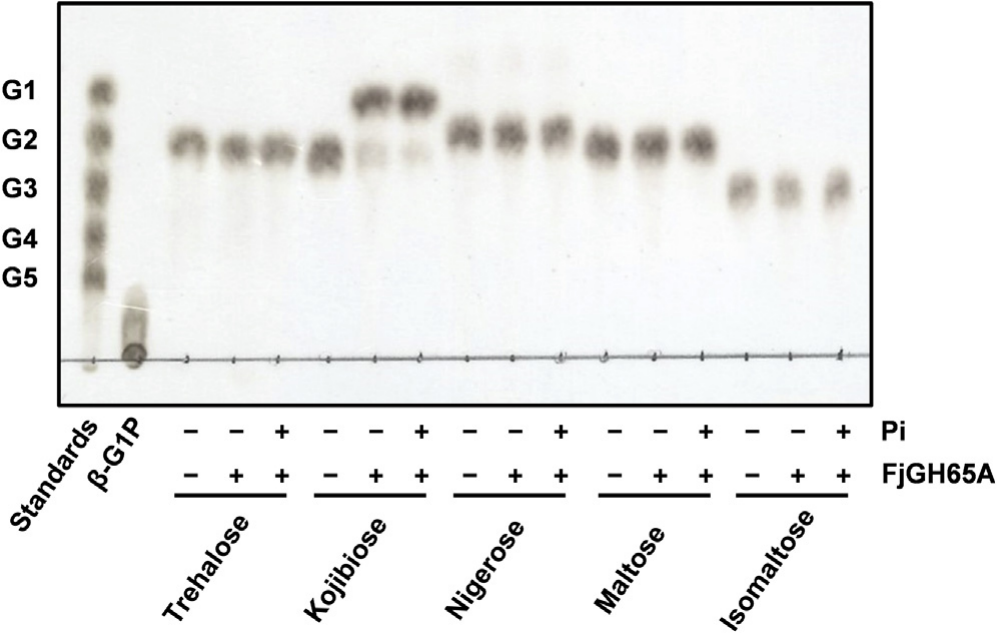
**Hydrolytic activity of FjGH65A to α-glucobiose.** FjGH65A was incubated with 10 mM α-glucobiose for 10 min, as described in the Experimental procedures section. The reaction products were then analyzed by TLC. β-G1P, β-glucose 1-phosphate; G1, glucose; G2, maltose; G3, maltotriose; G4, maltotetraose; G5, maltopentaose.

To determine the substrates of FjGH65A, we first examined its activity toward α-glucobiose because other known GH65 enzymes are active on α-glucosides. FjGH65A displayed activity against kojibiose and produced only glucose in the presence or absence of inorganic phosphate, but hydrolytic activity toward other α-glucobioses was not detected by TLC (Fig. 3). This result indicates that FjGH65A is not a GP but a GH specific for α-1,2-glucosidic linkage. Using the glucose oxidase–peroxidase method, we detected fainter hydrolytic activity for nigerose (0.063 ± 0.006 unit mg^−1^) than kojibiose (33.9 ± 1.2 unit mg^−1^); no activity was detected for the other disaccharides. We also found that FjGH65A hydrolyzed longer kojioligosaccharides, from kojitriose to kojipentaose, but a kinetic analysis showed that *k*_cat_/*K*_m_ values for longer kojioligosaccharides were lower than for kojibiose (Table 1). In addition, FjGH65A showed no activity toward *p*-nitrophenyl α-glucopyranoside, which is a general substrate of exo-acting α-glucoside hydrolases. The optimum pH and temperature of FjGH65A were 5.5 and 40 °C, respectively (Fig. S2, *A* and *B*). The enzyme was stable (>80% residual activity) up to 50 °C after 30 min incubation and in a pH range of 4.5 to 9.0 (Fig. S2, *C* and *D*).

**Table 1:** Kinetic parameters of FjGH65A

Enzyme	Substrate	*K*_m_ (mM)	*k*_cat_ (s^−1^)	*k*_cat_/*K*_m_ (s^−1^ mM^−1^)	Relative activity (%)^a^
FjGH65A	Kojibiose	0.28 ± 0.01	108 ± 0.1	399	100
	Kojitriose	0.13 ± 0.01	46.5 ± 0.1	273	68
	Kojitetraose	0.82 ± 0.03	67.5 ± 1.2	94.6	23
	Kojipentaose	0.96 ± 0.10	44.5 ± 1.6	46.3	11
	G2G6G	0.44 ± 0.02	72.8 ± 1.4	212	53
	G2G2G6G	0.83 ± 0.09	59.3 ± 2.2	71.5	18
	α-GlcF	3.1 ± 0.2	56.1 ± 1.8	18.1	4.5
YcjT^b^	Kojibiose	1.05	1.1	1.1	
TbKP^c^	Kojibiose	0.77	170	220	

a *k*_cat_/*K*_m_ value for kojibiose is taken as 100%.

b *Escherichia coli* K-12 kojibiose phosphorylase (51).

c *Thermoanaerobium brockii* ATCC 35047 kojibiose phosphorylase (17).

Dextran is a polymer of α-D-glucose coupled primarily with α-1,6 linkages produced by several bacteria, including *Leuconostoc* and *Streptococcu*s species, with varying amounts of side branches bound by α-1,2, α-1,3, or α-1,4 depending on the bacterial species and strain (34). FjGH65A showed weak but obvious activity (1.38 ± 0.04 unit mg^−1^) against *Leuconostoc citreum* NRRL B-1299 α-glucan, which contains many α-1,2-linked branches in the α-1,6-linked main chain (35). By contrast, FjGH65A did not hydrolyze commercial dextran (which is rarely branched), *L. citreum* NRRL B-1355 α-glucan, composed of alternating α-1,3 and α-1,6 linkages (36, 37) or soluble starch at all. Next, we hypothesized that FjGH65A could hydrolyze shorter oligosaccharides produced by the degradation of dextran-containing α-1,2 branches; therefore, 6-*O*-α-kojibiosylglucose (G2G6G) and 6-*O*-α-kojitriosylglucose (G2G2G6G) were enzymatically synthesized and used for analysis (see Supporting information). FjGH65A hydrolyzed G2G6G and G2G2G6G with *k*_cat_/*K*_m_ values that were comparable but slightly lower than those for kojitriose and kojitetraose, respectively (Table 1). However, the types of linkages of the reducing ends were different from kojioligosaccharides. These results suggest that FjGH65A specifically recognizes the Glcα-1,2-Glc moiety in oligosaccharides and has relatively relaxed recognition for the reducing end of the oligosaccharides.

### Anomeric configuration of products

**Figure 4:**
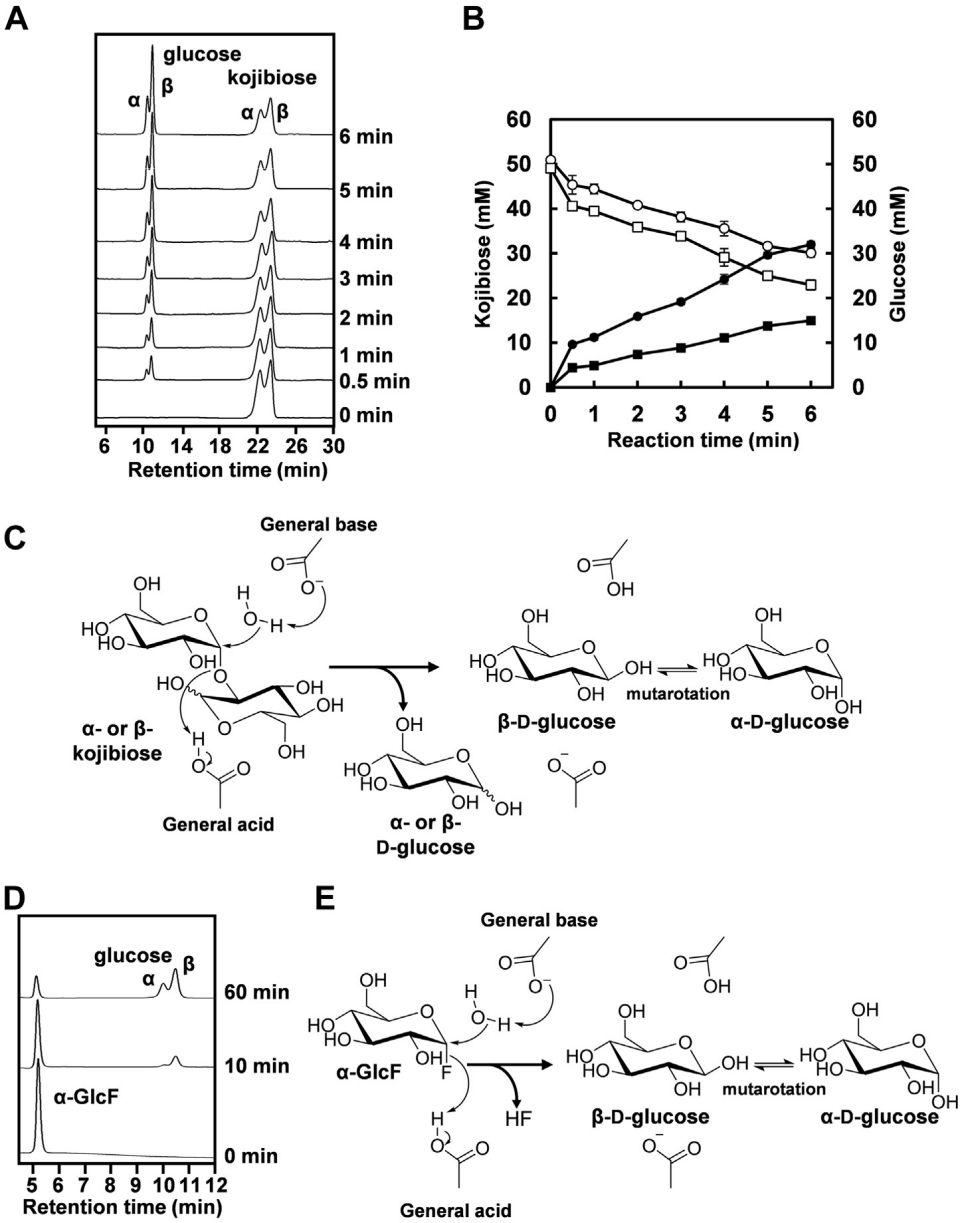
**Anomeric analysis of the hydrolytic products of FjGH65A.** HPLC profiles of the anomeric analysis of the hydrolysis of kojibiose (*A*) and α-GlcF (*D*) by FjGH65A. *B*, the concentrations of substrate α/β-kojibiose and product α/β-glucose during hydrolysis by FjGH65A. *Open squares*, α-kojibiose; *open circles*, β-kojibiose; *closed squares*, α-glucose; *closed circles*, β-glucose. The proposed reaction mechanism of FjGH65A on kojibiose (*C*) and α-GlcF (*E*).

To explore the catalytic mechanism of FjGH65A, the initial products of FjGH65A hydrolysis against kojibiose and α-D-glucopyranosyl fluoride (α-GlcF) were analyzed *via* normal-phase HPLC. This system can separate α-glucose and β-glucose (see Experimental procedures); the retention times of α-glucose and β-glucose were 10.0 and 10.5 min, respectively (Fig. S3). When unhydrolyzed kojibiose (0 min) was applied to the HPLC, two peaks were detected at retention times of 22 and 23 min with an area ratio of 49% and 51%, respectively (Fig. 4*A*). Considering the ratio of α-kojibiose and β-kojibiose in aqueous solution (38), the 22 min peak and the 23 min peak likely corresponded to α-kojibiose and β-kojibiose, respectively. In comparison with the chromatograms of the 0 and 0.5 min reactions, a small peak at a retention time of 10.0 min and a large peak at a retention time of 10.5 min appeared, whereas the peaks at retention times of 22 and 23 min decreased. The amounts of glucose and kojibiose were determined from the peak areas in the chromatograms (Fig. 4*B*). From the slopes of these plots, FjGH65A digested α-kojibiose and β-kojibiose at a ratio of 1:0.8 and produces α-glucose and β-glucose at a ratio of 1:2.2. This result suggests that FjGH65A hydrolyzes kojibiose *via* an anomer-inverting mechanism (Fig. 4*C*). However, because these products were derived from both the nonreducing end α-glucose and the reducing-end α/β-glucose of α/β-kojibiose, it was difficult to clearly distinguish between them. To further elucidate the reaction mechanism, α-GlcF (*k*_cat_ = 56.1 ± 1.8 s^−1^, *K*_m_ = 3.1 ± 0.2 mM) was used as a substrate. Similarly, β-glucose initially accumulated in the 10 min reaction, whereas α-glucose was produced with a delay as the hydrolysis reaction progressed (Fig. 4*D*). This result strongly supported the hypothesis that FjGH65A is an α-glucoside hydrolase with an inverting mechanism (Fig. 4*E*).

### Overall structure

**Figure 5:**
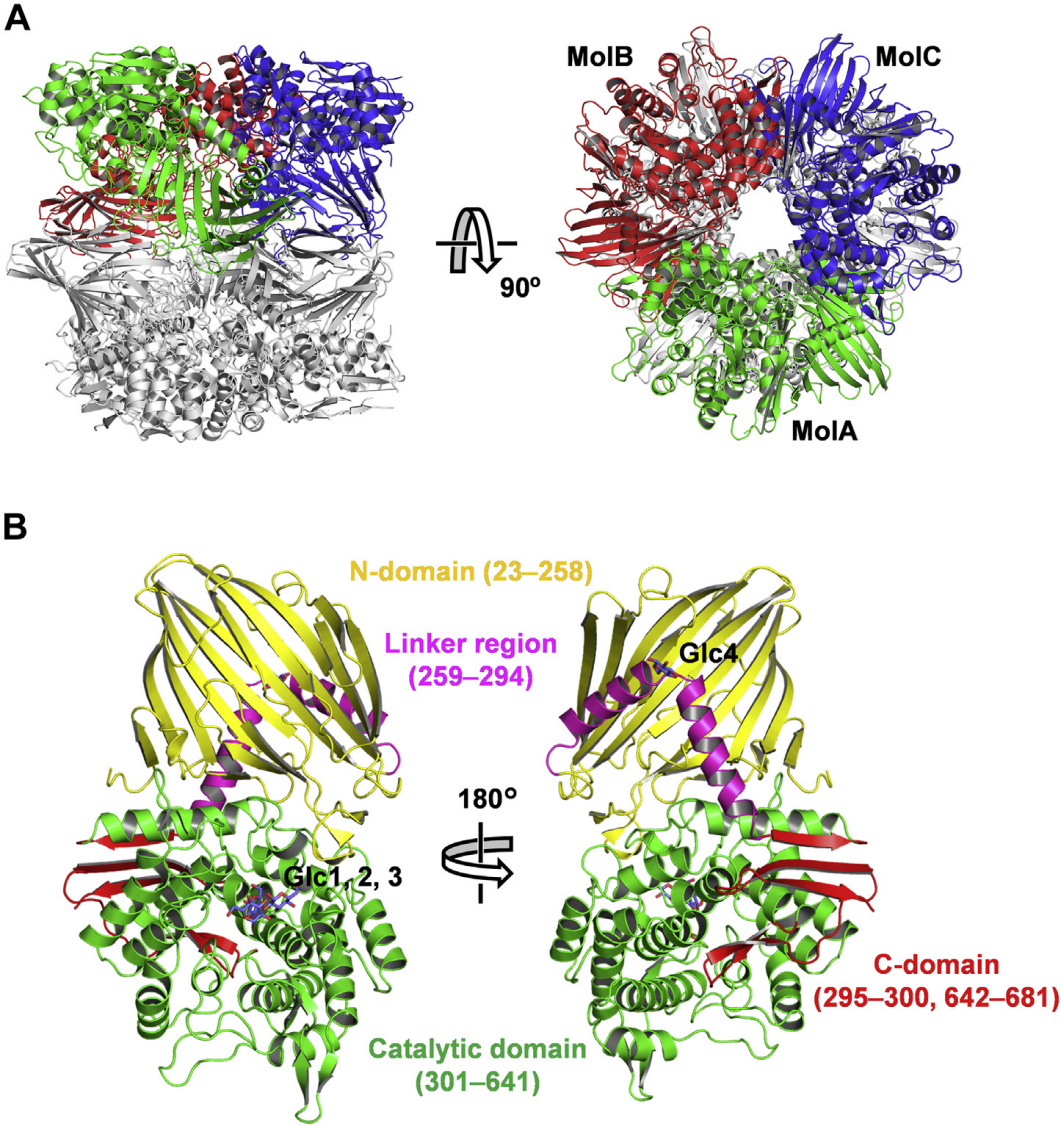
**The overall structure of FjGH65A.***A*, ribbon model of the FjGH65A hexamer. Three monomers, MolA, MolB, and MolC, forming a trimer are colored in *green*, *red*, and *blue*, respectively. The other trimer related by the crystallographic two-fold rotational axis is colored in *gray*. *B*, ribbon model of the FjGH65A monomer. The colors used are as follows: N-domain, *yellow*; linker region, *magenta*; catalytic domain, *green*; and C-domain, *red*. The bound glucose molecules (Glc1–Glc4) are indicated as *stick models* in *slate blue*.

The crystal structure of FjGH65A was solved using the single-wavelength anomalous dispersion method using a KAuCl_4_-soaked crystal because the molecular replacement method using the reported structures of GH65 GPs failed. We determined the structure of the enzyme in unliganded form and in complex with glucose at 1.54 and 1.40 Å resolutions, respectively (Table 2). Of all 681 amino acid residues, we were able to successfully model residues 23 to 681. The FjGH65A crystals belong to the space group *C*2 and contain three monomers (named MolA, MolB, and MolC) in the asymmetric unit. PISA (https://www.ebi.ac.uk/pdbe/pisa/) analysis showed that FjGH65A forms a “dimer of trimers” hexamer related by the crystallographic two-fold rotational axis (Fig. 5*A*). This result is consistent with the gel-filtration chromatography result described above. The total surface area of the hexamer is 123,460 Å^2^, whereas the buried interface area is 31,510 Å^2^. All GH65 enzymes of known structure were dimers, and the amino acid residues responsible for hexamer formation in FjGH65A were not conserved in any reported GH65 enzymes (27, 28). The monomer of FjGH65A comprises four regions as follows: an N-terminal β-sandwich domain (N-domain, residues 23–258), a helical linker region (residues 259–294), an (α/α)_6_-barrel catalytic domain (residues 301–641), and a C-terminal β-sheet domain (C-domain, residues 295–300 and 642–681) (Fig. 5*B*). A structural similarity search was then performed using the Dali server (39). GH65 enzymes such as kojibiose phosphorylase from *Caldicellulosiruptor saccharolyticus* [CsKP, Protein Data Bank (PDB) 3WIR, *Z*-score = 38.8], 2-*O*-α-glucosylglycerol phosphorylase from *Bacillus selenitireducens* (BsGGP, PDB 4KTP, *Z*-score = 37.1), and maltose phosphorylase from *Levilactobacillus brevis* (LbMP, PDB 1H54, *Z*-score = 35.6) showed a high degree of structural similarity with FjGH65A although their amino acid sequence identities were only 28%, 23%, and 19%, respectively. GH15 enzymes such as glucodextranase from *Arthrobacter globiformis* (PDB 1ULV, *Z*-score = 22.0) and glucoamylase from *Thermoanaerobacterium themosaccharolyticum* (TtGA, PDB 1LF6, *Z*-score = 21.7) also showed significant structural similarity despite low amino acid sequence identities (17% and 20%, respectively). Although the structure of the FjGH65A N-domain is similar to those of the reported GH65 GPs, a loop (residues 71–78) in the N-domain is shorter than the corresponding region (residues 62–79) in LbMP (27). The loop is located in the interface of the “dimer of trimers” and is suggested to be involved in the hexamer formation of the FjGH65A. The FjGH65A C-domain consists of five β-strands, which are fewer than those of the other GH65 GPs.

**Table 2:** Data collection and refinement statistics

Data	KAuCl_4_ derivative	Native	Glucose complex
Data collection			
Beamline	PF-AR NW12A	PF-AR NW12A	PF-AR NW12A
Wavelength (Å)	1.0402	1.0000	0.9795
Space group	*C*2	*C*2	*C*2
Unit cell			
*a* (Å)	121.3	122.8	123.5
*b* (Å)	194.8	194.0	194.2
*c* (Å)	110.4	111.7	112.0
*β* (º)	113.5	116.6	116.6
Resolution range (Å)	50–2.0 (2.11–2.00)	50–1.54 (1.62–1.54)	50–1.40 (1.48–1.40)
Total reflections	1,016,744	2,319,581	3,073,386
Unique reflections	154,913	342,453	451,842
Completeness (%)	98.2 (96.2)	99.7 (99.4)	98.0 (96.6)
*R*_merge_	0.087 (0.744)	0.050 (0.916)	0.060 (0.755)
*R*_meas_	0.095 (0.852)	0.54 (0.992)	0.065 (0.820)
*R*_pim_	0.038 (0.347)	0.021 (0.379)	0.025 (0.318)
CC_1/2_	0.999 (0.982)	0.999 (0.846)	0.999 (0.810)
*I*/*σ*	11.6 (1.9)	19.9 (2.3)	15.6 (2.4)
Redundancy	6.6 (6.4)	6.8 (6.7)	6.8 (6.5)
Refinement statistics			
Resolution (Å)		1.54	1.40
*R*_work_		0.170	0.156
*R*_free_		0.192	0.170
Number of atoms			
Protein (MolA, B, C)		5330, 5305, 5323	5300, 5280, 5282
Ligand (MolA, B, C)		40, 24, 40	48, 48, 48
Water		1452	1668
Mean *B* factor (Å^2^)			
Protein (MolA, B, C)		28.4, 35.4, 28.2	25.1, 27.4, 26.9
Ligand (MolA, B, C)		32.1, 35.5, 32.7	21.7, 24.7, 26.3
Water		35.4	32.5
RMSD			
Bond lengths (Å)		0.009	0.009
Bond angles (º)		1.501	1.526
Ramachandran plot			
Favored (%)		96.7	97.2
Outliers (%)		0	0
Clashscore		1.98	1.7
MolProbity score		1.16	1.06

### Active site of FjGH65A

**Figure 6:**
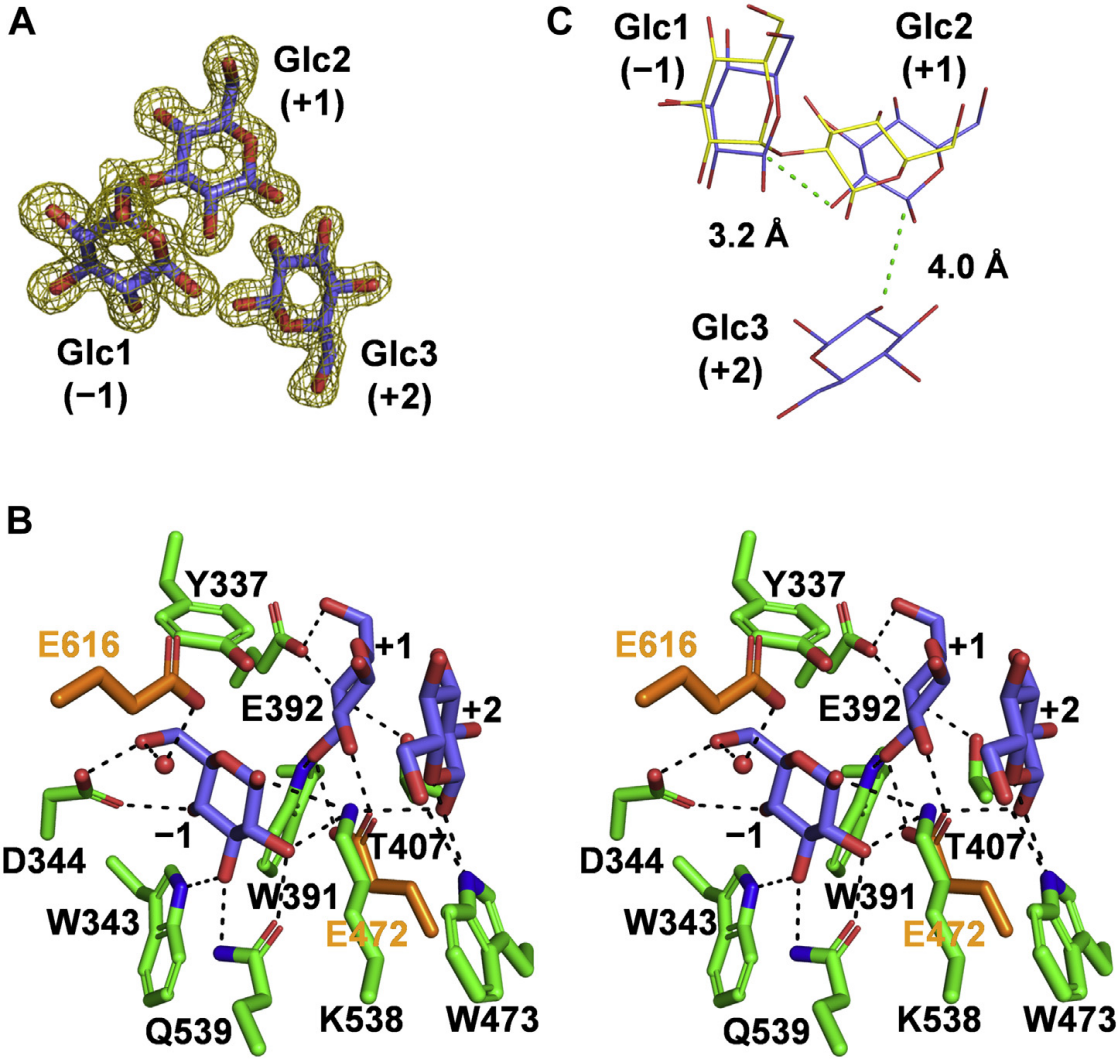
**Active site of FjGH65A complexed with glucose.***A*, *F*_*o*_*− F*_*c*_ omit electron density maps contoured at 3 *σ*, and the glucose models are shown as *olive mesh* and *slate blue stick* models, respectively. *B*, stereo view of the active site of FjGH65A. The side chains of amino acid residues surrounding the glucose are shown in *green stick* models, and the proposed catalytic residues and glucose are in *orange* and *slate blue*, respectively. The labels of the catalytic residues are highlighted in *orange*. The hydrogen bonds are represented by *black dashed lines*. A water molecule interacting with glucose and the base Glu^616^ are shown as a *red sphere*. The subsite numbers, according to Davies *et al.* (40), are also indicated. *C*, depicts the superimposition of ligands in the crystal structures of FjGH65A in complex with glucose and CsKP in complex with kojibiose (PDB 3WIQ). Glucose (*slate blue*) and kojibiose (*yellow*) are shown as *thin stick* models. The distances between the O2 atom of Glc2 and the C1 atom of Glc1 and between the O2 atom of Glc3 and the C1 atom of Glc2 are shown as *green dashed lines.* CsKP, kojibiose phosphorylase from *Caldicellulosiruptor saccharolyticus*.

During the refinement, the *F*_o_ − *F*_c_ electron density for β-glucose molecules was found in the glucose-complex structure of FjGH65A. Each monomer of FjGH65A binds four β-glucose molecules, three of which (named Glc1, Glc2, and Glc3) was observed at the center of the catalytic domain (Figs. 5*B* and 6*A*), whereas the other (Glc4) is bound to the linker region (Figs. 5*B* and S4). The following descriptions are based primarily on MolA. The *B* factors of Glc1, Glc2, Glc3, and Glc4 are 15.5, 14.7, 23.4, and 33.2 Å^2^, respectively. Glc1 is located at subsite −1 (subsite nomenclature is according to Davis *et al.* (40)) and interacts with the side chains of four amino acid residues (Trp^343^, Asp^344^, Lys^538^, and Gln^539^) *via* hydrogen bond (Fig. 6*B*). Glc2 is located at subsite +1 and forms hydrogen bonds with Trp^391^, Glu^392^, Thr^407^, and Glu^472^. Glc3 at subsite +2 interacts with the side chain of Trp^473^ *via* fewer hydrogen bonds and is partially exposed to the solvent (Fig. 6*B*). It is unclear how kojioligosaccharide substrates bind the active site, but the reducing-end of substrates is thought to protrude out into the solvent. These observations are suggested to be consistent with the fact that the activity against longer kojioligosaccharides is lower than that against kojibiose. Similarly, GH97 α-glucoside hydrolase SusB prefers maltotriose to longer maltooligosaccharides because of less interaction with the substrate at subsite +3, which is open to solvent (41). Among the hydroxy groups of Glc2, the O2 atom of Glc2 is the closest to the C1 atom of Glc1 (distance = 3.2 Å) (Fig. 6*C*). The superposition of the glucose-complex and CsKP in complex with kojibiose (PDB 3WIQ) demonstrates that the orientation of Glc2 is similar to that of the reducing-end glucose of kojibiose in CsKP. Among the hydroxy groups of Glc3, the O2 atom of Glc3 is the closest to the C1 atom of Glc2 (distance = 4.0 Å).

**Figure 7:**
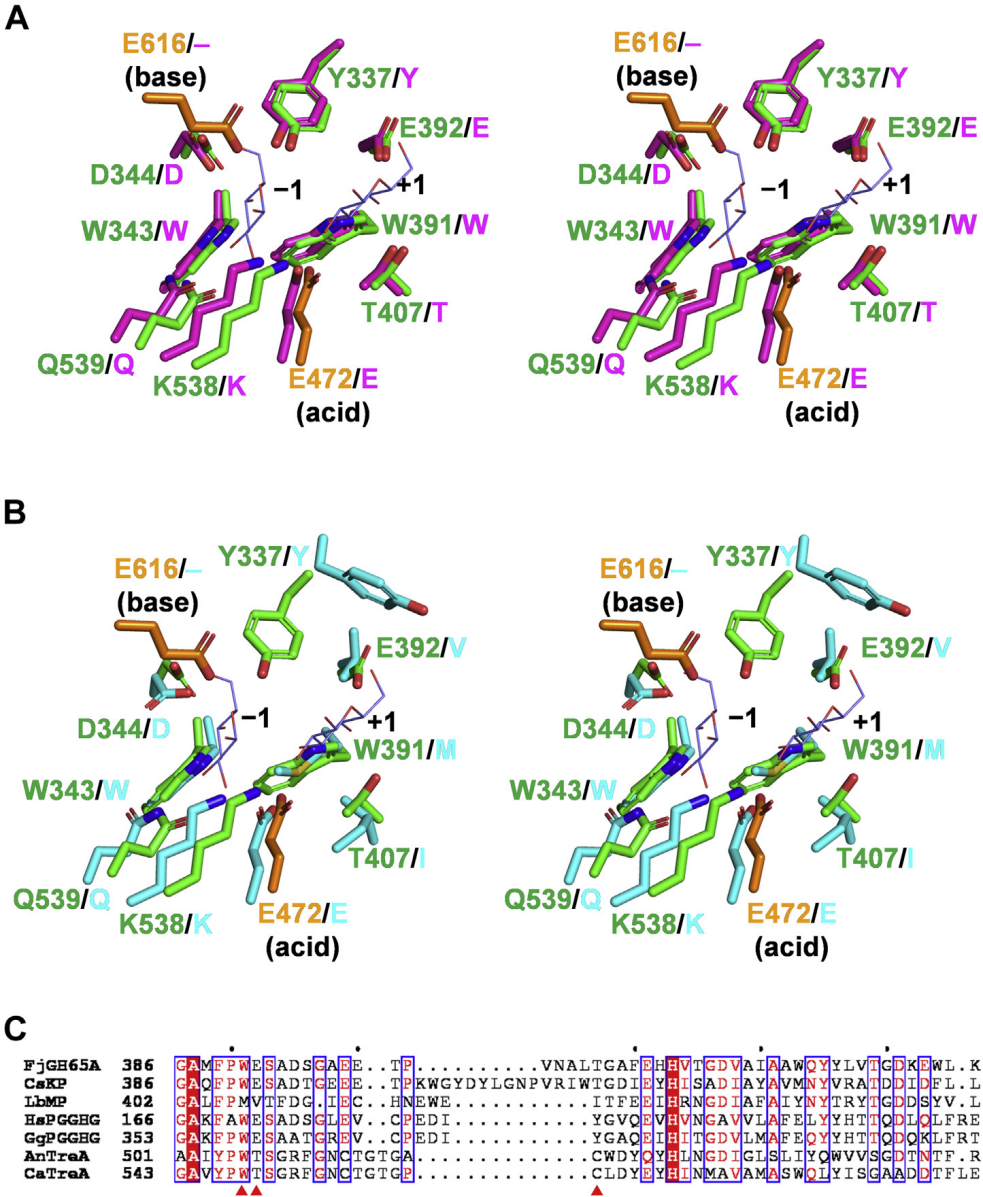
**Comparison of the active sites of GH65 enzymes and FjGH65A.***A* and *B*, superimposition of the active sites of FjGH65A (*green*), CsKP (PDB 3WIQ, *magenta*) (*A*), and LbMP (PDB 1H54, *cyan*) (*B*) in stereo. Glucose (*slate blue*) is shown as a *thin stick* model. The catalytic residues of FjGH65A are shown in *orange*. *C*, sequence alignments of the region containing Trp^391^, Glu^392^, and Thr^407^ of FjGH65A and its corresponding regions in GH65 GPs and GHs. The amino acid residues corresponding to Trp^391^, Gln^362^, and Thr^407^ are indicated by *red triangles*. The full sequence alignment is shown in Fig. S5. AnTreA, *Aspergillus nidulans* trehalase; CaTreA, *Candida albicans* trehalase; CsKP, kojibiose phosphorylase from *Caldicellulosiruptor saccharolyticus*; GgPGGHG, *Gallus gallus* PGGHG; GH65, glycoside hydrolase family 65; HsPGGHG, *Homo sapiens* PGGHG; LbMP, maltose phosphorylase from *Levilactobacillus brevis*; PGGHG, Protein α-glucosyl-1,2-β-galactosyl-L-hydroxylysine α-glucosidase.

**Figure 8:**
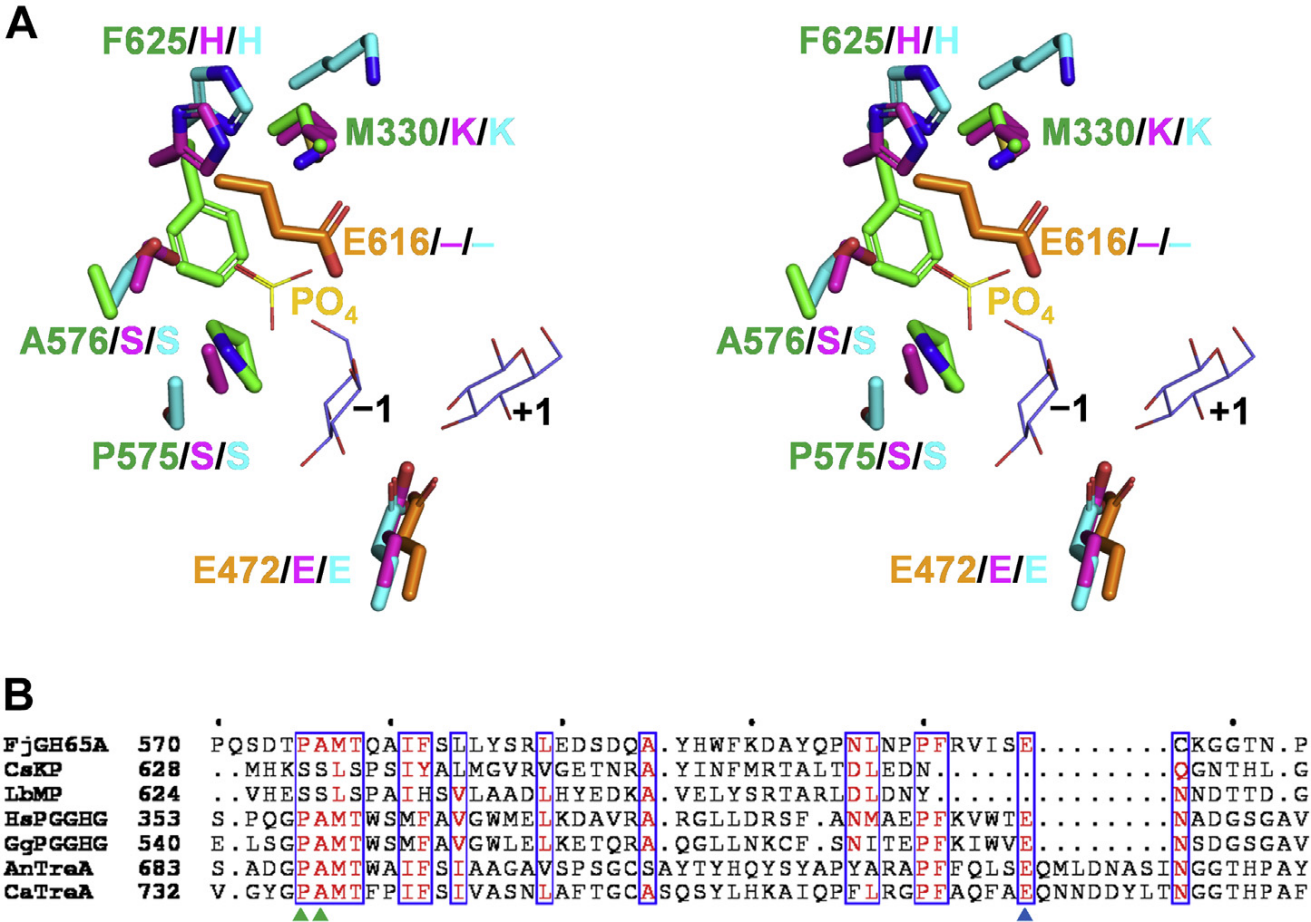
**Comparison of the phosphate-binding sites of GH65 GPs and FjGH65A.***A*, superimposition of the phosphate-binding sites of CsKP (PDB 3WIR, *magenta*), LbMP (PDB 1H54, *cyan*), and FjGH65A (*green*) in stereo. Glucose (*slate blue*) and phosphate (*yellow*) are shown as *thin stick* models. The catalytic residues are colored in *orange*. *B*, depicts the sequence alignment of the regions around the phosphate-binding sites of GH65 GPs and their corresponding regions in GH65 GHs, including FjGH65A, vertebrate PGGHGs, and fungal acid trehalase. The amino acid residues corresponding to the serine residues bound to phosphate in GH65 GPs and the general base residue of FjGH65A are indicated by *green* and *blue triangles*, respectively. The full sequence alignment is shown in Fig. S5. AnTreA, *Aspergillus nidulans* trehalase; CaTreA, *Candida albicans* trehalase; CsKP, kojibiose phosphorylase from *Caldicellulosiruptor saccharolyticus*; GgPGGHG, *Gallus gallus* PGGHG; GH65, glycoside hydrolase family 65; GPs, glycoside phosphorylases; HsPGGHG, *Homo sapiens* PGGHG; LbMP, maltose phosphorylase from *Levilactobacillus brevis*; PGGHG, Protein α-glucosyl-1,2-β-galactosyl-L-hydroxylysine α-glucosidase.

The active sites of CsKP in complex with kojibiose (PDB 3WIQ) and LbMP in native form (PDB 1H54) were superposed onto the FjGH65A active site. The amino acid residues interacting with Glc1, as well as the general acid Glu^472^, are conserved in both CsKP and LbMP, whereas the amino acid residues surrounding Glc2 (Trp^391^, Glu^392^, and Thr^407^ in FjGH65A) are conserved in CsKP (which acts on the same substrate as FjGH65A) but not in LbMP (Fig. 7, *A* and *B*). The sequence alignment of FjGH65A and the reported eukaryotic GH65 GHs, including fungal acid trehalases and PGGHGs, indicate that the subsite −1 residues are completely conserved whereas the subsite +1 residues are different from each other (Fig. 7, *B* and *C*). All the GH65 GPs whose structure has yet been determined to possess a phosphate-binding site consisting of lysine, histidine, and two serine residues (27, 28). In CsKP in complex with glucose and phosphate (PDB 3WIR), a phosphate molecule makes polar interactions with the two serines and is surrounded by histidine and lysine (Fig. 8*A*). In FjGH65A, the two serine residues are replaced by Pro^575^ and Ala^576^, and the histidine and lysine residues are replaced by Phe^625^ and Met^330^ (Fig. 8, *A* and *B*). Instead, FjGH65A has Glu^616^ in the sterically similar position as the phosphate-binding site in GH65 GPs. Glu^616^ is located in a good position to act as a general base, and Glu^472^ is conserved as a general acid among GH65 GHs and GPs. We constructed the mutants E472Q and E616Q, where each glutamic acid residue was substituted with glutamine; both the mutants lost activity (<0.1% of wild type) toward kojibiose. Based on the primary structure alignment, Glu^616^ is completely conserved among the GH65 GHs (Fig. 8*B*), suggesting that Glu^616^ acts as a general base on FjGH65A and is therefore essential for hydrolytic reactions.

### Comparison with clan GH-L enzymes

**Figure 9:**
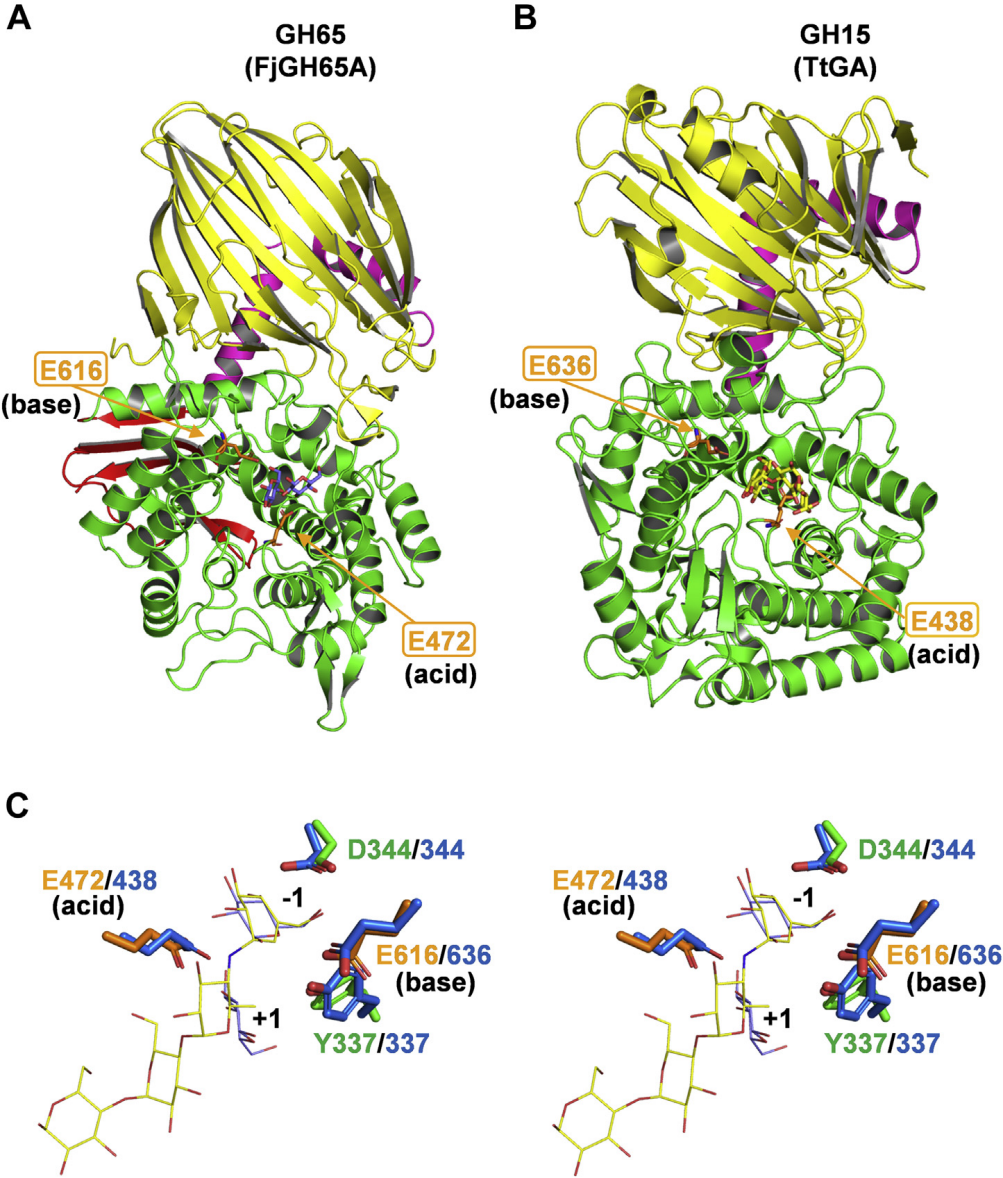
**Structural comparison of FjGH65A with a structural homolog belonging to GH15.***A* and *B*, the overall structures of FjGH65A in complex with glucose (*A*) and GH15 glucoamylase TtGA (PDB 1LF9) form *T. themosaccharolyticum* in complex with acarbose (*B*) are shown. The catalytic residues are indicated as *orange stick* models. *C*, stereo view of the active sites of FjGH65A and TtGA. The color description for FjGH65A is the same as in Figure 8. The overlaid residues of TtGA and acarbose are shown in *blue* and *yellow*, respectively. GH, glycoside hydrolase; GH65, glycoside hydrolase family 65; TtGA, glucoamylase from *Thermoanaerobacterium themosaccharolyticum*.

GH15 and GH65 share an anomer-inverting mechanism and an (α/α)_6_-barrel catalytic domain. Based on these similarities, they are classified into clan GH-L and have been thought to have a related evolutionary origin (27). The overall structure of FjGH65A was compared with that of GH15 glucoamylase TtGA in complex with acarbose (PDB 1LF9). FjGH65A and TtGA share an N-domain, a linker region, and an (α/α)_6_-barrel catalytic domain (Fig. 9, *A* and *B*). Figure 9*C* shows the superimposition of Cα atoms of catalytic residues and conserved residues in FjGH65A and TtGA. The general acid and base catalysts are located on the loops between the α5 and α6 helices and between the α11 and α12 helices of the (α/α)_6_-barrel catalytic domain and are structurally conserved. In TtGA, Asp^344^ is located at the α2 helix and contributes to the capture of nucleophilic water molecules *via* hydrogen bonding with the O6 of glucose bound to subsite −1 (42). Tyr^337^ is located between the α1 and α2 helices of TtGA and forms a hydrogen bond with the general base Glu^636^ and is highly conserved in GH15 (43). In the superposition, the side chains of these residues overlap each other; Glc1 in FjGH65A and the cyclohexene of acarbose in TtGA also overlap at subsite −1 (Fig. 9*C*).

## Discussion

**Figure 10:**
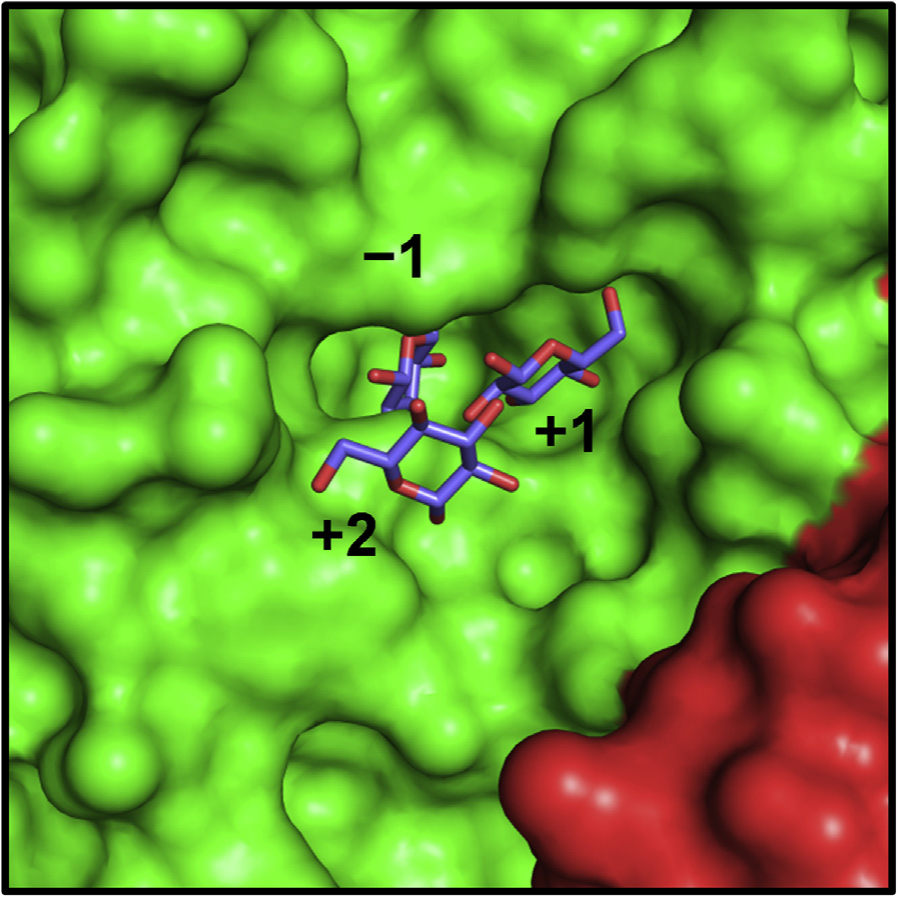
**Surface model of the FjGH65A active site.** MolA and MolB are colored in *green* and *red*, respectively. Glc1, Glc2, and Glc3 are colored in *slate blue*.

In this study, we elucidated the enzymatic function and three-dimensional structure of FjGH65A. FjGH65A has a strict specificity for α-1,2-glucosidic linkages and efficiently hydrolyzed kojibiose. This disaccharide is present in very small amounts in *koji* extract, *sake*, starch hydrolysates, among other products (44, 45, 46) and shows a prebiotic effect on beneficial colonic bacterial species such as *Bifidobacteria* (47). Although several GHs that hydrolyze kojibiose have been reported, all of these also hydrolyze other α-1,X-glucosides (41, 48, 49, 50). FjGH65A has high affinity and high catalytic efficiency for kojibiose and kojitriose when compared with GH65 kojibiose phosphorylases (Table 1) (17, 51). Although FjGH65A showed low activity on α-1,2-branched dextran from *L. citreum* B-1299, it exhibited higher activity toward G2G6G, which is a substructure of α-1,2-branched dextran, with an affinity and catalytic efficiency following those for kojibiose and kojitriose. On this enzyme, subsite +2 is more spacious than subsites −1 and +1, which strictly recognize kojibiose, suggesting that oligosaccharides with α-linkages other than α-1,2 at the reducing end, such as G2G6G, are acceptable (Fig. 10). Therefore, it is likely that FjGH65A plays a role in the degradation of the oligosaccharides that are products resulting from the hydrolysis of α-1,2-branched dextran by peripheral gene products such as dextranase FjDex31A and/or a putative GH66 dextranase (52). FjGH65A has a signal peptide, indicating that the enzyme is periplasmic or extracellular. However, the metabolic pathway of α-1,2-branched dextran in *F. johnsoniae* must still be investigated further.

Several enzymes have been reported to specifically hydrolyze α-1,2-glucosidic linkages. Mannosyl-oligosaccharide glucosidase (EC 3.2.1.106), which is involved in *N*-glycan processing in the endoplasmic reticulum, hydrolyzes the Glcα-1,2-Glc unit in *N*-glycan precursors (53, 54). However, this enzyme does not hydrolyze kojibiose but is instead inhibited by it (54, 55). PGGHG can hydrolyze kojibiose, but its activity is only one-fifth of the activity against α-glucosyl-1,2-β-galactosyl-L-hydroxylysine (56). Branched-dextran exo-1,2-α-glucosidase (EC 3.2.1.115) specifically hydrolyzes α-1,2-glucosidic branches in *L. citreum* B-1299 α-glucan and produces only glucose. However, this enzyme has been reported not to hydrolyze kojibiose (57, 58). A GH65 GP known as BsGGP has been reported to cleave kojibiose at a slow rate in the presence of phosphate to produce two glucose molecules. This reaction is not direct hydrolysis and progresses depending on the phosphate: BsGGP phosphorolyzes kojibiose to form β-D-glucose 1-phosphate, which is then hydrolyzed by the same enzyme to produce glucose and inorganic phosphate (26). Thus, FjGH65A is a GH with a different substrate specificity from previously reported GHs that are active on α-1,2-glucosidic linkages. We here propose α-1,2-D-glucoside glucohydrolase as the systematic name and α-1,2-glucosidase as the short name for FjGH65A.

Although the enzymatic properties of GH65 GHs, including fungal acid trehalases and vertebrate PGGHGs, have been reported, the general base had not been identified, and the catalytic mechanism had not been investigated. The general base residue was predicted only by mutational analysis of human PGGHG (32). Using kojibiose and synthetic α-GlcF, we identified the hydrolytic mechanism of FjGH65A as an inverting mechanism, which to our knowledge is the first time this has been done for a GH65 GH. The crystal structure and mutational analysis of FjGH65A revealed that Glu472 and Glu616 are the catalytic acid and base, respectively, and support this mechanism. In addition, it is suggested that GH15 enzymes and GH65 GHs have common catalytic machinery with sterically conserved residues, including the catalytic acid and base residues. This reinforces the theory that GH15 and GH65 share a common ancestral protein. For example, GH130 contains inverting GPs and GHs active on β-mannosides and is the only inverting GH family where the structures of both GPs and GHs have been reported to date (59, 60). In GH130 β-mannoside phosphorylases, the basic amino acid residues that interact with inorganic phosphate are conserved (60, 61). By contrast, GH130 β-mannosidases have two glutamic acid residues that are expected to be involved in the hydrolysis at the position corresponding to the phosphate-binding site in GH130 β-mannoside phosphorylases (59). In addition, the amino acid residues forming subsite −1 are conserved between GH130 β-mannoside phosphorylase and β-mannosidase (59, 61). These features resemble the relationship between FjGH65A and the GH65 GPs.

Recently, the Conserved Unique Peptide Patterns program, a program for the functional annotation and subgrouping of proteins based on a new peptide-based similarity assessment algorithm, has been created for CAZymes (62, 63). GH65 is grouped by the Conserved Unique Peptide Patterns from GH65:1.1 to GH65.16.1. FjGH65A is among these and is classified as GH65:8.1, where GH65 proteins from *Elizabethkingia* and *Bacteroides* also belong. The glutamate residues proposed as the general acid and general base were conserved, and amino acid residues that form a phosphate-binding site are not present in GH65:8.1. Tryptophan and glutamate residues corresponding to Trp^391^ and Glu^392^ of FjGH65A, which are important for the recognition of kojibiose, were also conserved in GH65:8.1. Therefore, it may be the case that GH65 enzymes classified as GH65:8.1 may actually be α-1,2-glucosidases.

In conclusion, FjGH65A is the first bacterial GH65 GH and is a novel inverting α-1,2-glucosidase that specifically hydrolyzes kojibiose, unlike previously reported enzymes. We propose α-1,2-D-glucoside glucohydrolase as the systematic name and α-1,2-glucosidase as the short name for FjGH65A. Moreover, its crystal structure was determined as the first GH65 GHs and revealed the structural commonalities and differences involved in the catalysis and substrate recognition in GH65 GHs and GPs. Our results will help us to better understand the mechanisms of hydrolysis and substrate specificity in GH65 enzymes—including eukaryotic GHs—and to predict the functions of uncharacterized enzymes within not only GH65, but also other GH families that contain GPs.

## Experimental procedures

### Materials

The reagents used were of analytical grade and were purchased from FUJIFILM Wako Pure Chemicals or Nacalai Tesque unless otherwise noted. Kojibiose was purchased from Carbosynth, and isomaltose and β-glucose-1-phosphate were purchased from Tokyo Chemical Industries. Kojitriose, kojitetraose, kojipentaose, G2G6G, and G2G2G6G were synthesized by reverse phosphorolysis of kojibiose phosphorylase (Caur_2019) from *Chloroflexus aurantiacus* J-10-fl (see Supporting information, Figs. S6 and S7). α-Glucans from *L. citreum* NRRL B-1299 and *L. citreum* NRRL B-1355 (35, 36, 37) were kindly provided by Dr Mikihiko Kobayashi. α-GlcF was prepared by deacetylation of 2,3,4,6-tetra-*O*-acetyl-α-D-glucopyranosyl fluoride (Merck Millipore).

### Recombinant protein production and purification

FjGH65A (GenBank ID, ABQ07432.1) was initially predicted to have no signal sequence using the SignalP server (www.cbs.dtu.dk/services/SignalP/) (64) although the sequence contains some hydrophobic amino acid residues at the N-terminus. We found another candidate for the start codon of FjGH65A 39-bp upstream, whose translation product was predicted to have a signal sequence of 23 amino acid residues, consistent with the sequence found in the RefSeq database (WP_044048041.1). The amino acid residues are numbered according to WP_044048041.1 in this article. The gene for FjGH65A (ABQ07432.1, residues 14–681) was amplified from *F. johnsoniae* NBRC 14942 (ATCC 17061, UW101) by colony-direct PCR using KOD FX Neo DNA polymerase (Toyobo) and a pair of primers, FjGH65AΔ13-NheI-F and FjGH65A-XhoI-R (Table S1). The amplified gene product was then ligated into a pET28a (+) vector (Merck Millipore) using the NheI and XhoI restriction sites. The resulting plasmid was used as a template for inverse PCR with a pair of primers, FjGH65A-F and FjGH65A-R (Table S1), to construct the expression plasmid of the N-terminally His-tagged FjGH65A without the signal sequence (residues 24–681). Site-directed mutagenesis was performed *via* inverse PCR with the desired primers (Table S1) using the recombinant FjGH65A expression plasmid as a template. The sequences of the constructs were verified by DNA sequencing. *E. coli* BL21(DE3) cells harboring the expression plasmid were grown in LB (1% tryptone, 0.5% yeast extract, and 1% NaCl) medium supplemented with 50 μg/ml kanamycin (Merck Millipore) at 37 °C until the absorbance reached 0.6 to 0.8. Isopropyl β-D-1- (Merck Millipore) was added at a final concentration of 0.1 mM, and the culture medium was incubated at 20 °C for 24 h. After induction, the cells in 500 ml of the culture medium were collected, resuspended in 30 ml of 20 mM Tris-HCl (pH 7.5) containing 300 mM NaCl and 20 mM imidazole, and sonicated for 15 min. The supernatant after centrifugation (20,640*g*, 4 °C, 30 min) was applied to a Ni-Sepharose excel (GE Healthcare) column equilibrated with the same buffer. The column was washed with buffer and the recombinant proteins were eluted with 20 mM Tris-HCl (pH 7.5) containing 300 mM NaCl and 100 to 250 mM imidazole. The 250 mM imidazole-eluted fraction was concentrated in 20 mM sodium citrate buffer (pH 6.0) containing 150 mM NaCl by ultrafiltration using an Amicon Ultra 30,000 molecular cut off filter (Merck Millipore), further purified *via* gel-filtration chromatography using an ÄKTA explorer system (GE Healthcare) with a HiPrep 16/60 Sephacryl S-200 HR column (GE Healthcare) and eluted with the same buffer. The column was calibrated with the following standards: thyroglobulin (669 kDa), ferritin, (440 kDa), aldolase (158 kDa), conalbumin (75 kDa), and ovalbumin (44 kDa). Protein purity was determined by SDS-PAGE. The marker used was the ExcelBand All Blue Broad Range Plus Protein Marker (SMOBiO). Enzyme concentrations were calculated using ExPASy ProtParam (http://web.expasy.org/protparam/) using molar absorption coefficients (FjGH65A, 18,640 M^−1^ cm^−1^) calculated by measuring the absorbance at 280 nm with a NanoDrop 2000c (Thermo Fisher Scientific).

### Enzyme assays

When α-glucobiose was used as a substrate, the hydrolysis activity of FjGH65A was analyzed *via* TLC with the following reaction conditions: 10 mM substrate and 10 μg/ml (130 nM) purified FjGH65A at 30 °C for 10 min. The reaction solution and authentic standards (glucose, maltooligosaccharides, and β-glucose-1-phosphate) were spotted on TLC aluminum sheet silica gel 60 F254 and developed with 1-butanol:ethanol:water = 5:5:2. To calculate the specific activity of FjGH65A against trehalose, kojibiose, nigerose, maltose, isomaltose, kojitriose, kojitetraose, kojipentaose, dextran 40,000, dextran 200,000, B-1299 α-glucan, B-1355 α-glucan, and soluble starch, release glucose was quantified using the glucose oxidase–peroxidase method using a Glucose C-II Test Kit (Wako Pure Chemical). The enzyme reaction volume was a 50 μl reaction mixture containing 100 μg/ml (1.3 μM) (against α-glucobioses except for kojibiose), 1 μg/ml (13 nM) (against kojibiose), or 50 μg/ml (650 nM) (against other substrates) of purified FjGH65A, 1 mM oligosaccharide or 1% (w/v) polysaccharide, and 50 mM sodium citrate buffer (pH 5.5) at 30 °C. After incubation for 30 min, the reactions were stopped by boiling for 5 min. Because nigerose is degraded by heat treatment under neutral conditions (65), the reaction was stopped by adding 0.5 M Na_2_CO_3_ when nigerose was used as a substrate. One unit of FjGH65A activity was defined as the amount of enzyme that hydrolyzed 1 μmol of α-glucosidic bond per minute.

To determine the optimal pH, the enzyme reaction was carried out in McIlvaine buffer (pH 3.0–8.0) containing 1 μg/ml (13 nM) FjGH65A at 30 °C for 10 min and 1 mM kojibiose as a substrate. To determine the optimal temperature, the enzymatic reaction was carried out in 50 mM sodium citrate buffer (pH 5.5) containing 10 μg/ml (130 μM) FjGH65A, 20 mM sodium citrate buffer (pH 6.0), 150 mM NaCl, and 1 mM kojibiose as a substrate for 10 min at 25 to 70 °C. To measure pH stability, 900 μg/ml (11.7 μM) FjGH65A was incubated at 4 °C for 17 h in 100 mM sodium citrate buffer (pH 3.5–6.0), 100 mM sodium phosphate buffer (pH 6.0–9.0), or 100 mM glycine-NaOH buffer (pH 9.0–11.0). After incubation, the concentration of FjGH65A was diluted to 100 μg/ml (1.3 μM) with 100 mM sodium citrate buffer (pH 5.0). To measure temperature stability, 100 μg/ml (1.3 μM) FjGH65A was incubated at 4 to 60 °C for 30 min in 20 mM sodium citrate buffer (pH 6.0) containing 150 mM NaCl. All the reactions were stopped at the appropriate time by boiling for 5 min. The remaining activity was measured in 50 μl reaction mixtures that contained 1 μg/ml (13 nM) of treated FjGH65A, 1 mM kojibiose, and 50 mM sodium citrate buffer (pH 5.5) at 30 °C for 10 min.

### Kinetics studies

The initial velocities of the hydrolytic reactions for kojioligosaccharides, G2G6G, and G2G2G6G were determined using the 50 mM sodium citrate buffer (pH 5.5) and at least four concentrations of substrates were used, that is, 0.1 to 2 mM kojibiose, 0.1 to 2 mM kojitriose, 0.2 to 3 mM kojitetraose, 0.2 to 5 mM kojipentaose, 0.2 to 2 mM G2G6G, and 0.2 to 5 mM G2G2G6G. The enzyme concentrations used were 1 μg/ml (13 nM) against kojibiose, kojitriose, kojitetraose, and G2G6G or 10 μg/ml (130 nM) against kojipentaose and G2G2G6G. The amount of liberated glucose was quantified *via* the glucose oxidase–peroxidase method by using a Glucose C-II Test Kit. The same procedure was performed three times for each reaction. The reaction kinetics parameters were determined *via* nonlinear regression analysis implemented by KaleidaGraph (Synergy Software).

### Analysis of the anomeric form of the product

Anomers of the hydrolytic products of kojibiose and α-GlcF were analyzed *via* normal-phase HPLC. The enzymatic reaction was performed in a 50 mM sodium citrate buffer (pH 5.5) at 30 °C containing 100 mM of each substrate and 100 μg/ml (1.3 μM) FjGH65A. The reactions were carried out for 30 s, 1 min, 2 min, 3 min, 4 min, 5 min, and 6 min when kojibiose was used as the substrate; the reactions were carried out for 10 min, 35 min, 60 min, and 180 min when α-GlcF was used as the substrate. The reaction mixtures were then applied to a TSK-GEL amide-80 column (4.6 × 250; Tosoh) immediately after incubation and were eluted with 80% (v/v) acetonitrile at a flow rate of 1.2 ml/min at 25 °C. The reaction products were detected using a refractive index detector (RID-10A, Shimadzu). The retention times of α-glucose (Merck Millipore) and β-glucose (Tokyo Chemical Industry) were determined in the same manner.

### Crystallization and structure determination

FjGH65A (30 mg/ml in 20 mM sodium citrate buffer, pH 6.0 and 150 mM NaCl) was crystallized at 20 °C by the hanging drop vapor diffusion method; 1 μl of protein solution was mixed with an equal volume of the mother liquor consisting of 12% (w/v) PEG3350 (Hampton Research), 0.3 M ammonium citrate buffer (pH 7.0), and 10 mM tris (2-carboxyethyl) phosphine hydrochloride (Hampton Research). The crystals were cryoprotected with a reservoir solution supplemented with 20% (v/v) ethylene glycol or 30% (w/v) glucose and quickly frozen in liquid nitrogen. For phase determination, the crystals were soaked in a reservoir solution supplemented with 10 mM KAuCl_4_ at 20 °C for 16 h before cryoprotection. Diffraction data were collected at the NW12 A beamline (Photon Factory). The data were first processed *via* XDS (66) and then scaled using SCALA (67) as implemented in the CCP4 package (68). The initial phase was determined *via* single-wavelength anomalous dispersion using a single crystal soaked in KAuCl_4_ and the phase determination program Phaser (69) on CCP4. The unliganded structure of FjGH65A and the complex structure with glucose were determined using the molecular replacement program MOLREP (70). Manual model building was performed using COOT (71), and refinement was performed using REFMAC5 (72) and Translation/Libration/Screw Motion Determination (73). Molecular images were made using PyMOL (Schrödinger LLC). Structural similarity searches were performed using the Dali server (39). Table 2 summarizes the data collection and refinement statistics.

### Sequence alignment and phylogenetics

The protein sequences were aligned using Clustal Omega (74), and the figures were generated using ESPript 3.0 (75). For phylogenetic analysis, the protein sequences were aligned using MUSCLE (76), and the resulting alignment was used for generating a phylogenetic tree *via* the maximum likelihood method using MEGA 7 (77).

## Data availability

The atomic coordinates and structure factors have been deposited in the Worldwide Protein Data Bank (http://wwpdb.org/) under accession codes 7FE3 and 7FE4. All other data are contained within the article.

## Supporting information

This article contains supporting information.

## Conflict of interest

The authors declare that they have no conflicts of interest with the contents of this article.
