# Supplementary material for: Structure of a bacterial α-1,2-glucosidase defines mechanisms of hydrolysis and substrate specificity in GH65 family hydrolases
Source: J Biol Chem. 2021 Oct 30;297(6):101366. doi: 10.1016/j.jbc.2021.101366 (PMC8626586; doi:10.1016/j.jbc.2021.101366)
Supplement: Supplementary Methods, Figures S1–S7 and Table S1 [file mmc1.pdf]

Supporting information

Structure of a bacterial α-1,2-glucosidase defines mechanisms of hydrolysis and substrate specificity in GH65 family hydrolases

Shuntaro Nakamura (中村 駿太郎) [1], Takanori Nihira (仁平 高則) [2], Rikuya Kurata (倉田 陸矢) [3], Hiroyuki Nakai (中井 博之) [2], Kazumi Funane (舟根 和美) [4], Enoch Y. Park (朴 龍洙) [1,3,5], and Takatsugu Miyazaki (宮崎 剛亜) [1,3,5*]

[1] Department of Bioscience, Graduate School of Science and Technology, Shizuoka University, Shizuoka, Japan
[2] Faculty of Agriculture, Niigata University, Niigata, Japan
[3] Department of Agriculture, Graduate School of Integrated Science and Technology, Shizuoka University, Shizuoka, Japan.
[4] Faculty of Life and Environmental Sciences, University of Yamanashi, Kofu, Yamanashi, Japan
[5] Research Institute of Green Science and Technology, Shizuoka University, Shizuoka, Japan.

***Correspondence:** Takatsugu Miyazaki, Research Institute of Green Science and Technology, Shizuoka University, 836 Ohya, Suruga-ku, Shizuoka, 422-8529, Japan; Tel.: +81-54-238-4886; E-mail: miyazaki.takatsugu@shizuoka.ac.jp

List of materials included

Supplementary Methods

Figure S1. Expression and purification of FjGH65A.

Figure S2. Effects of pH and temperature on FjGH65A hydrolysis.

Figure S3. Electron density map of Glc4.

Figure S4. HPLC analysis of glucose anomer.

Figure S5. Sequence alignment of GH65 enzymes

Figure S6. TLC analysis of the products prepared by reverse phosphorolysis using kojibiose as the acceptor.

Figure S7. TLC analysis of the products prepared by reverse phosphorolysis using isomaltose as the acceptor.

Table S1. Sequence of oligonucleotides used in this study.

Supplementary Methods

Kojitriose, kojitetraose, kojipentaose, 6-*O*-α-kojibiosylglucose (G2G6G), and 6-*O*-α-kojitriosylglucose (G2G2G6G) were synthesized by reverse phosphorolysis of kojibiose phosphorylase (Caur_2019) using β-D-glucose 1-phosphate (β-Glc1P) as the donor and kojibiose or isomaltose as the acceptor.

The gene *caur_2019* (GenBank ID: ABY35234) was amplified by PCR from the genomic DNA of *Chloroflexus aurantiacus* J-10-fl using KOD-plus DNA polymerase (TOYOBO Co., Ltd., Osaka, Japan) with the following oligonucleotides based on the genomic sequence (GenBank accession number CP000909.1): 5′-ggaattccatatgtggatacttgctgaac-3′ as the forward primer, containing an NdeI site (underlined) and 5′-tttctcgagcgcagtggtgtaacgtcc-3′ as the reverse primer, containing an XhoI site (underlined). The amplified *caur_2019* was purified using a FastGene Gel/PCR Extraction Kit (NIPPON Genetics Co., Ltd., Tokyo, Japan), before digestion with NdeI and XhoI (New England Biolabs, Beverly, MA, USA), and insertion into pET24a (+) (Merck KGaA, Darmstadt, Germany) to encode a His_6 tag fusion at the C-terminus of the recombinant protein. The expression plasmid was propagated in *Escherichia coli* DH5α (TOYOBO Co., Ltd.) and was purified by a FastGene Plasmid Mini Kit (NIPPON Genetics Co., Ltd.) before verification by sequencing (Eurofins Genomics K.K., Tokyo, Japan). An *E. coli* BL21 (DE3) (Merck KGaA) transformant harboring the expression plasmid was grown at 37°C in 200 mL Luria–Bertani medium (1% tryptone, 0.5% yeast extract, and 0.5% NaCl) containing 50 μg/mL of kanamycin until the absorbance of the growth solution reached 0.6 at 600 nm. Expression was induced by 0.1 mM isopropyl β-D-1-thiogalactopyranoside and continued at 18°C for 24 h. The cells were then harvested by centrifugation at 4,000 × g for 20 min and then suspended in 50 mM HEPES-NaOH buffer (pH 7.5) containing 500 mM NaCl (buffer A). The suspended cells were disrupted by sonication (Ultrasonic Generator Model 201M, KUBOTA Co., Tokyo, Japan). The supernatant was collected via centrifugation at 20,000 × g for 20 min and applied to a HisTrap FF column (Cytiva, Marlborough, MA, USA), equilibrated with buffer A containing 10 mM imidazole, using ÄKTA prime (Cytiva). After washing with buffer A, which contained 22 mM imidazole, and subsequent elution using a 22–400 mM imidazole linear gradient in buffer A, the fractions containing the recombinant protein were pooled, dialyzed against 10 mM HEPES-NaOH buffer (pH 7.0), and concentrated (AMICON Ultra-15 filter; Merck KGaA). The protein concentration was determined spectrophotometrically at 280 nm using a theoretical extinction coefficient of $\varepsilon = 164210$ cm^{-1} M^{-1} on the basis of the amino acid sequence. Caur_2019 phosphorolyzed kojibiose but not trehalose, nigerose, or maltose, suggesting that it is a kojibiose phosphorylase.

Kojitriose, kojitetraose, and kojipentaose were prepared via reverse phosphorolysis of Caur_2019 using kojibiose as the acceptor; 20 mL of the reaction mixture (pH 6.5) containing 500 mM β-Glc1P, 500 mM kojibiose, and 0.57 mg/mL Caur_2019 was incubated at 37°C for 120 h (Fig. S6). After terminating the reaction via heat treatment at 80°C for 30 min and deionization via electrodialysis using a Micro Acilyzer S1 (ASTOM Co., Tokyo, Japan) with AC-220-10 (molecular weight cut-off of 300) (ASTOM Co.), the products were then purified using a Toyopearl HW-40S column (5 cm internal diameter × 100 cm; Tosoh Co., Tokyo, Japan) and equilibrated with MilliQ water (at a flow rate of 4 mL/min). Each fraction containing kojitriose, kojitetraose, and kojipentaose was collected and lyophilized. The amounts of kojitriose, kojitetraose, and kojipentaose obtained were 42, 13, and 32 mg, respectively.

G2G6G and G2G2G6G were prepared by reverse phosphorolysis of Caur_2019 using isomaltose as the acceptor. The 2 mL reaction mixture (pH 6.5) containing 500 mM β-Glc1P, 500 mM isomaltose, and 0.57 mg/mL Caur_2019 was incubated at 37°C for 65 h (Fig. S7). The reaction was stopped by adding 1 mL of 1 M HCl and incubating for 2 h. After neutralization by adding 1 mL of 1 M NaOH and deionization by electrodialysis, the products were purified using a Toyopearl HW-40S column equilibrated with MilliQ water. Each fraction containing G2G6G and G2G2G6G was collected and lyophilized. The amounts of G2G6G and G2G2G6G obtained were 62 and 36 mg, respectively.

The structures of these oligosaccharides were confirmed via NMR spectra taken in D_2O.

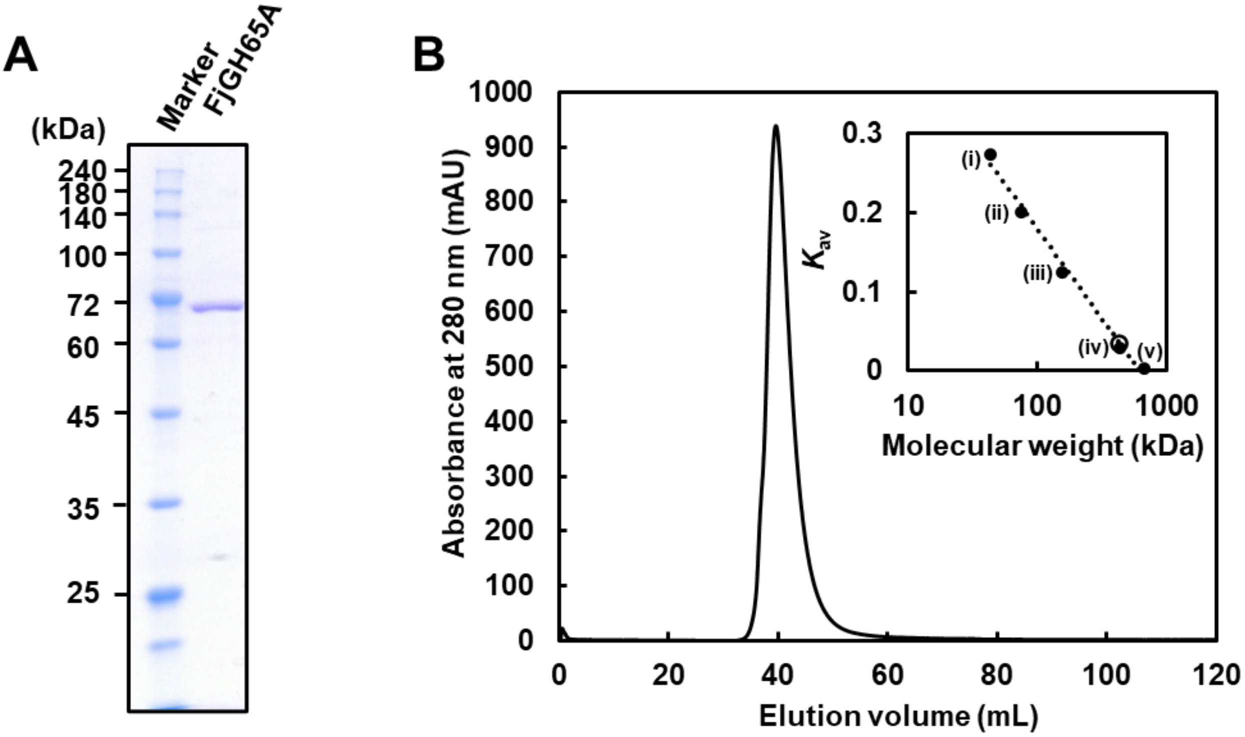

Figure S1. Expression and purification of FjGH65A.

(A) SDS-PAGE analysis of purified FjGH65A. The molecular weight marker used is ExcelBand All Blue Broad Range Plus Protein Marker PM1700 (SMOBIO Technology, Hsinchu City, Taiwan). (B) A chromatogram and calibration curve for molecular weight estimation of FjGH65A via gel filtration chromatography. Calibration was performed using ovalbumin (44 kDa, i), conalbumin (75 kDa, ii), aldolase (158 kDa, iii), ferritin (440 kDa, iv), and thyroglobulin (669 kDa, v). Protein standards and FjGH65A are indicated as filled and open circles.

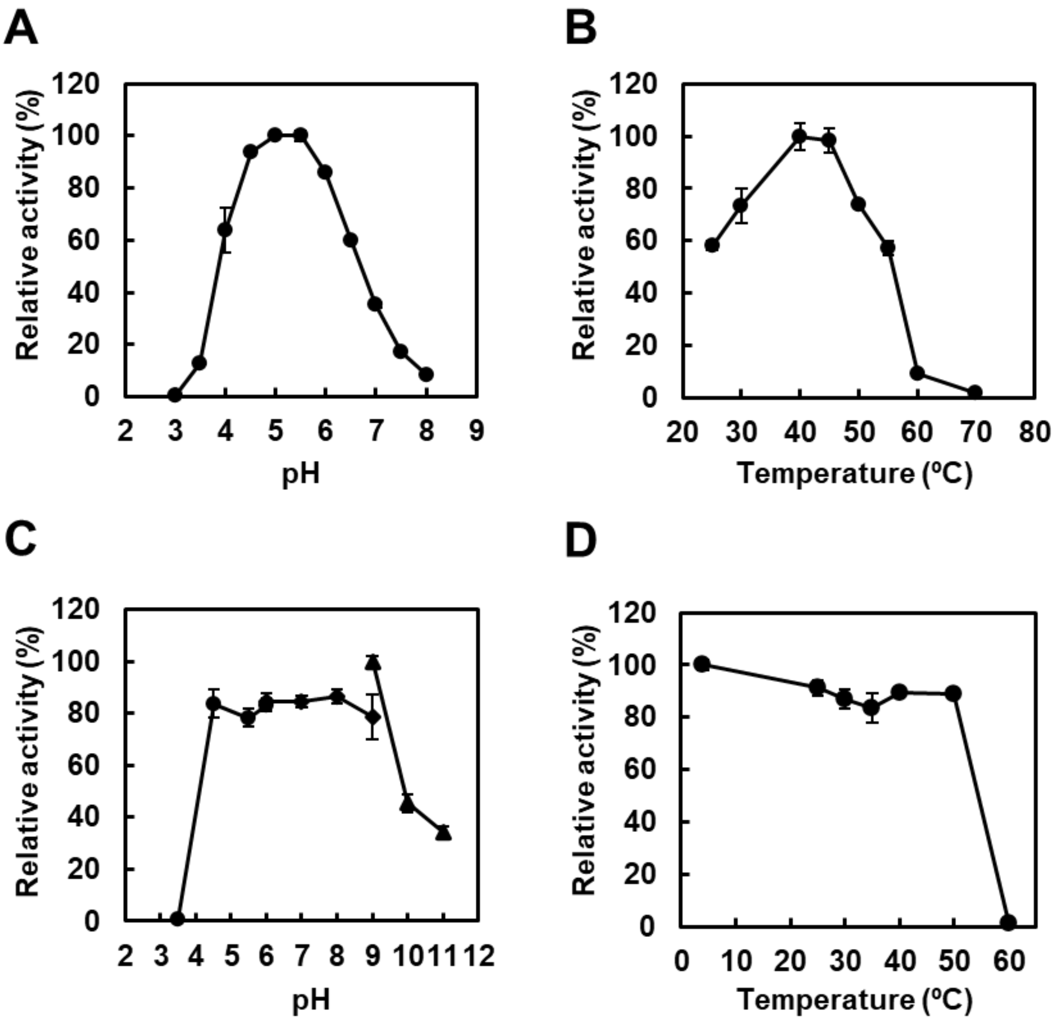

Figure S2. Effects of pH and temperature on FjGH65A hydrolysis.

(A and B) pH dependence (A) and temperature dependence (B) of FjGH65A hydrolysis for kojibiose. pH dependence was measured at 30°C for 10 min using McIlvaine buffer (pH 3.0–8.0). Temperature dependence was measured at 25°C–80°C using 50 mM sodium citrate buffer (pH 5.5). (C and D) pH stability (C) and temperature stability (D) of FjGH65A. For pH stability, the enzyme was incubated for 17 h in 50 mM sodium citrate buffer (pH 3.5–6.0, shown as circles), 50 mM sodium phosphate buffer (pH 6.0–9.0, shown as diamonds), or 50 mM glycine-NaOH buffer (pH 9.0–11.0, shown as triangles) at 4°C. For temperature stability, the enzyme was incubated for 30 min in 20 mM sodium citrate buffer (pH 6.0) containing 150 mM NaCl at 4°C–60°C. The residual activity was measured at 30°C for 10 min after each incubation.

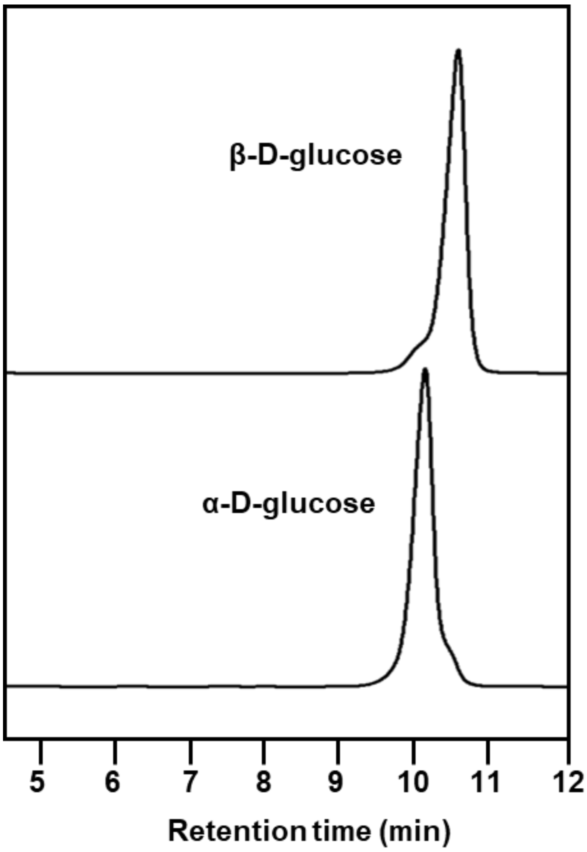

Figure S3. HPLC analysis of glucose anomer.

Separation of pyranose anomers of D-glucose by normal-phase HPLC using a TSK-GEL amide-80 column (4.6 × 250; Tosoh, Tokyo, Japan) immediately after dissolution. Sugars were eluted with 80% (v/v) acetonitrile at a flow rate of 1.2 mL/min at 25°C.

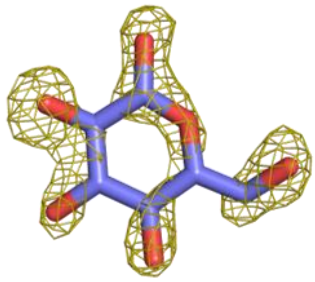

Figure S4. Electron density map of Glc4.

The F_o−F_c omit electron density map contoured at 3 σ and the glucose model are shown as *olive mesh* and a *slate blue* stick diagram, respectively.

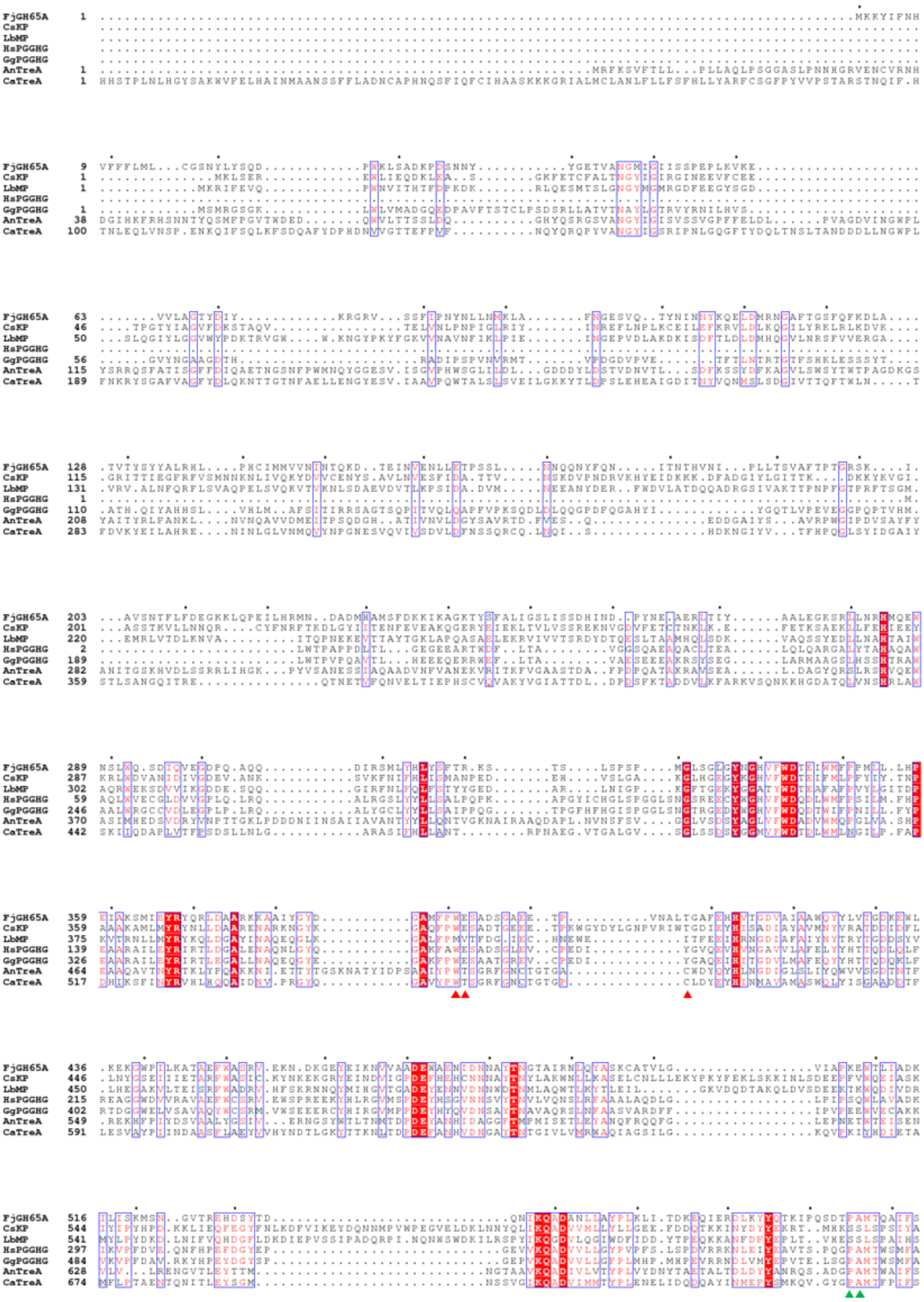
FjGH65A
CsKP
LbMP
HsPGGHG
GgPGGHG
AnTreA
CaTreA

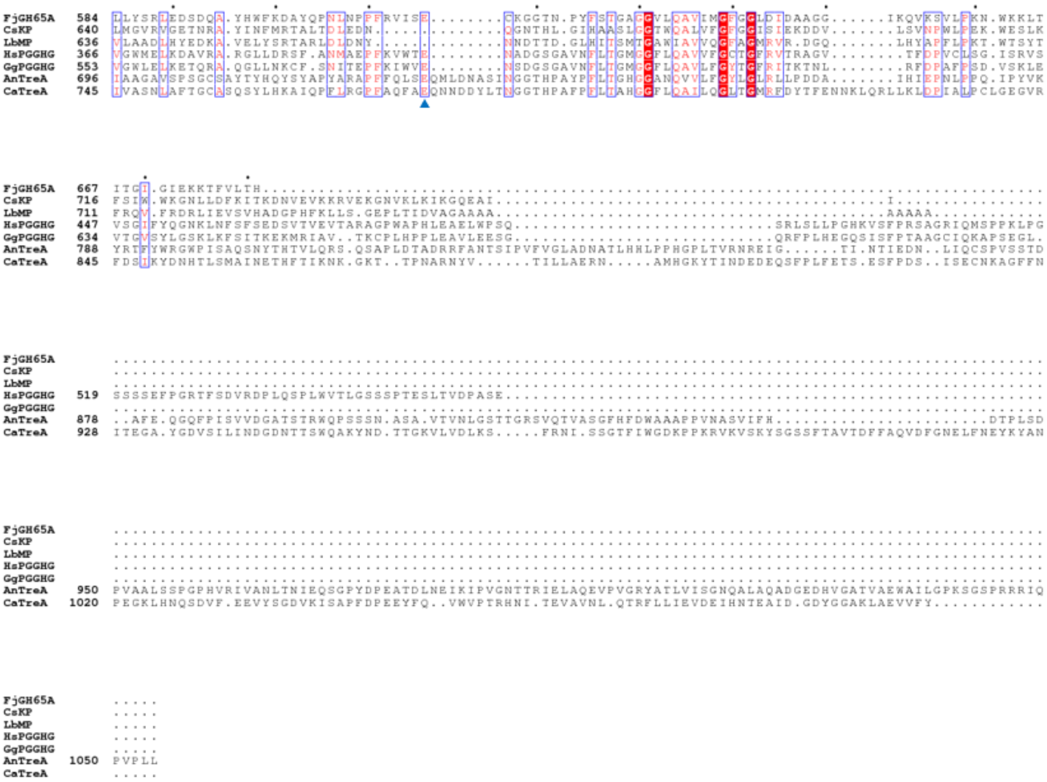

Figure S5. Sequence alignment of GH65 enzymes

The sequences of GH65 enzymes, including FjGH65A, bacterial GPs, vertebrate PGGHGs, and fungal acid trehalase were aligned using Clustal Omega. A figure was then generated using ESPript 3.0. Abbreviations: CsKP, *Caldicellulosiruptor saccharolyticus* KP; LbMP, *Levilactobacillus brevis* MP; HsPGGHG, *Homo sapiens* PGGHG; GgPGGHG, *Gallus* PGGHG; AnTreA, *Aspergillus nidulans* trehalase; *Candida albicans* trehalase. Amino acid residues corresponding to Trp^{391}, Gln^{362}, and Thr^{407}, the catalytic base residue of FjGH65A, and serine residues that are essential for phosphate binding are indicated as *red, blue,* and *green* triangles.

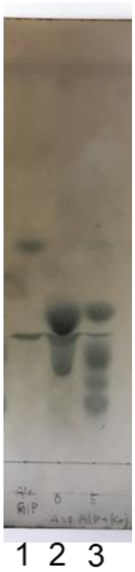

Figure S6. TLC analysis of the products prepared by reverse phosphorolysis using kojibiose as the acceptor.

Lane 1 represents 10 mM glucose + β-Glc1P as markers. Lanes 2 and 3 represent 10-fold diluted mixtures before and after the enzymatic reaction, respectively. The samples were spotted on TLC plates (Kieselgel 60 F254; 5 × 7.5 cm, Merck KGaA), and the plates were developed using a mobile phase of 80 % acetonitrile in water. The TLC plate was soaked in a solution of sulfuric acid/*p*-anisaldehyde/acetic acid/ethanol (18:13:5:478, by volume) and heated in an oven to detect the compounds.

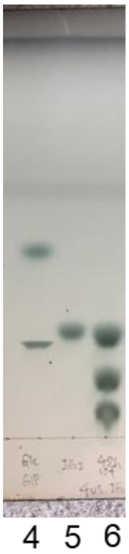

Figure S7. TLC analysis of the products prepared by reverse phosphorolysis using isomaltose as the acceptor.

Lanes 4 and 5 represent 10 mM glucose + β-Glc1P and 10 mM isomaltose as markers, respectively. Lane 6 represents a 10-fold diluted mixture after enzymatic reaction using isomaltose as an acceptor. The samples were spotted on TLC plates (Kieselgel 60 F254; 5 × 7.5 cm, Merck KGaA), and the plates were developed using a mobile phase of 80 % acetonitrile in water. The TLC plate was soaked in a solution of sulfuric acid/*p*-anisaldehyde/acetic acid/ethanol (18:13:5:478, by volume) and heated in an oven to detect the compounds.

Table S1. Sequence of oligonucleotides used in this study.

Oligonucleotide	Sequence (5'→3') [a],
FjGH65AΔ13-NheI-F	TTTGCTAGCCTATGCGGCAGCAATTATTTG
FjGH65A-XhoI-R	TTTTCTCGAGTTAATGAGTTAGTACAAAAGTC
FjGH65A-F	GCAGCCATCAGGATCCGTGGAAGCTT
FjGH65A-R	GATCCTGATGGCTGCCGCGCGGCAC
E472Q-F	TCGTTGCAGCAGATCAATGGGCTGAAAATA
E472Q-R	TATTTTCAGCCCATTGATCTGCTGCAACGA
E616Q-F	TCAGGGTCATTTCACAATGCAAGGGAGGGA
E616Q-R	TCCCTCCCTTGCATTGTGAAATGACCCTGA

[a] Restriction sites are underlined.
